# Occupational health risk assessment methods in China: A scoping review

**DOI:** 10.3389/fpubh.2022.1035996

**Published:** 2022-11-17

**Authors:** Lifang Zhou, Panqi Xue, Yixin Zhang, Fang Wei, Jiena Zhou, Shasha Wang, Yong Hu, Xiaoming Lou, Hua Zou

**Affiliations:** ^1^Institute of Occupational Health and Radiation Protection, Zhejiang Provincial Center for Disease Control and Prevention, Hangzhou, China; ^2^School of Medicine, Hangzhou Normal University, Hangzhou, China; ^3^Department of Public Health, Zhejiang University School of Medicine, Hangzhou, China; ^4^Shaoxing Center for Disease Control and Prevention, Shaoxing, China

**Keywords:** occupational health, risk assessment, qualitative, quantitative, semi-quantitative, scoping review

## Abstract

**Background:**

Over the decades, many assessment methods have been developed around the world and used for occupational health risk assessment (OHRA). This scoping review integrated the literature on methodological studies of OHRA in China and aimed to identifies the research hot-spots and methodological research perspectives on OHRA in China.

**Methods:**

A scoping review of literature was undertaken to explore the research progress on OHRA methods in China. Focusing on OHRA methods, the authors systematically searched Chinese and English databases and relevant guideline websites from the date of establishment to June 30, 2022. Databases included Web of Science, PubMed, Scopus, the China National Knowledge Internet, WanFang Database. Some other websites were also searched to obtain gray literature. The extracted information included the author, year, region of first author, the target industry, risk assessment model, study type, the main results and conclusions.

**Results:**

Finally, 145 of 9,081 studies were included in this review. There were 108 applied studies, 30 comparative studies and 7 optimization studies on OHRA in China. The OHRA methods studied included: (1) qualitative methods such as Romanian model, Australian model, International Council on Mining and Metals model, and Control of Substances Hazardous to Health Essentials; (2) quantitative methods such as the U. S. Environmental Protection Agency inhalation risk assessment model, Physiologically Based Pharmacokinetic, and Monte Carlo simulation; (3) semi-quantitative methods such as Singapore model, Fuzzy mathematical risk assessment model, Likelihood Exposure Consequence method and Occupational Hazard Risk Index assessment method; (4) comprehensive method (Chinese OHRA standard GBZ/T 298-2017). Each of the OHRA methods had its own strengths and limitations. In order to improve the applicability of OHRA methods, some of them have been optimized by researchers.

**Conclusions:**

There is a wide range of OHRA methods studied in China, including applied, comparative, and optimization studies. Their applicability needs to be further tested through further application in different industries. Furthermore, quantitative comparative studies, optimization studies, and modeling studies are also needed.

## Introduction

China is the most populous country in the world with a population of 1.41 billion, of which more than 783 million are workers, and more than 200 million workers are exposed to occupational hazards (1, 2). China has carried out a series of strategies and measures to reduce the health risk of occupational hazards exposure. However, occupational health in China still faces severe conditions and challenges (3). In 2021, China's National Health Commission (NHC) reported a total of 15,407 new cases of various occupational diseases nationwide (4). Occupational pneumoconiosis, noise-related hearing loss, and occupational poisoning have become the most serious occupational diseases in China (4, 5). In China, occupational diseases come from more than 30 industries, including traditional industries such as coal mining, non-ferrous metal mining, metallurgy, machinery, construction and chemical industry, and new industries such as computer and information technology, biology and medicine (3). Occupational diseases are reported in all provinces in China, but there are differences in distribution between different regions, for example, occupational tumors are predominant in Guangdong, Shandong, Liaoning, Hubei, Beijing, and Jiangsu (6), while chronic benzene poisoning is predominant in Guangdong, Jiangsu, Shandong, Beijing, Tianjin, Fujian, Zhejiang, and Sichuan (7, 8). Like many countries, China faces the important task of occupational health risk management in order to reduce the impact of occupational hazards. Occupational health risk assessment (OHRA) is an important part of occupational health management. Understanding how much exposure to a hazard poses health risks to workers is important to appropriately eliminate, control, and reduce those risks (9). The “Law on Prevention and Control of Occupational Disease,” which approved by the Chinese National People's Congress Standing Committee in 2002 and recently modified in 2018, stipulates that occupational health risk assessment is one of the tasks of the health administrative department in China (10).

Risk assessment is a process that aims to identify what hazards exist in the workplace and evaluating the possibility of personal injury or harm caused by these hazards. The purpose of risk assessment is to determine and propose corresponding preventive and control measures (11). OHRA is a process of qualitatively or quantitatively evaluating occupational health risk levels by comprehensively and systematically identifying and analyzing risk factors and protective measures in the workplace, so as to take corresponding controls (12). The use of risk assessment methods to evaluate the effects of toxic chemicals had its primary origin in 1976 when the U.S. Environmental Protection Agency (EPA) (13) adopted a methodology introduced in the 1950s to conduct the evaluation of suspect carcinogens (14, 15). In 1983, the U.S. Nuclear Regulatory Commission (16) first proposed that the risk assessment process is divided into four stages: hazard identification, dose-response assessment, exposure assessment and risk characterization (16). Since then, the risk assessment techniques and methods for health risks caused by hazardous substances have been widely used. Subsequently, the U.S. EPA established a series of risk assessment guidelines, such as the supplemental inhalation risk assessment guidelines of the Human Health Risk Assessment Manual, to provided technical guidance for the risk assessment of airborne toxic chemicals in the workplace (13).

Meanwhile, some European countries, Australia, Singapore, and other countries and organizations established guidelines for OHRA and risk management in response to occupational hazards in the workplace. For example, the United Kingdom Health and Safety Executive initiated the Control of Substances Hazardous to Health (COSHH) essentials control banding strategy (17); Australia has established risk management methods in Australian Standards (AS/NZS) (18); Romania established the risk assessment method for occupational accidents and diseases with reference to European standards (EN292/1-19, EN 1050/96) in 1998 (19); Singapore established guidelines for the hazard assessment of occupational chemical exposures for hazardous chemicals (20).

China established a classification standard for hazardous operations in the 1980s and started health risk assessment research by introducing the USEPA models in the nuclear industry field (12). In the first decade of OHRA study, the health risk assessment technology in China is mainly based on the four-step process recommended by the USEPA, focusing on monitoring of exposure and epidemiological study (21). In 2007, the “Technical Guidelines for Pre-Assessment of Occupational Disease Hazards in Construction Projects” (GBZ/T196-2007) promulgated by the Chinese Ministry of Health proposed that the risk assessment methods should be applied to the assessment of occupational disease hazards in construction projects (22). In 2010, the Chinese Ministry of Health issued standards such as “Classification for Hazards of Occupational Exposure to Toxicant” (GBZ 230-2010) and “Classification of Occupational Hazards at workplaces” (GBZ/T 229-2010) to rank hazard levels from exposure to productive dust, chemical toxicants, heat and noise (23, 24). Due to the lack of OHRA method in China, the GBZ 230-2010 and GBZ/T 229-2010 were sometimes used as an alternative to occupational health risk assessment, although the results are often not accurate enough due to the low sensitivity of the method.

In recent years, Chinese government has paid more and more attention to occupational health risk assessment. Researchers in China have applied some international risk assessment methods for occupational health risk assessment in various industries (21). At the same time, some researchers improved and optimized the OHRA tools introduced from abroad, and established the first Chinese OHRA standard “Guidelines for Occupational Health Risk Assessment of Chemicals in the Workplace” (GBZ/T 298-2017) (25), which recommends a quantitative risk assessment method, a qualitative risk assessment method and three semi-quantitative risk assessment methods. As China's first OHRA guideline, researchers have carried out applied studies on it in different industries soon after it was released (26, 27). The application of OHRA methods in GBZ/T 298-2017 shows that it still needs further improvement and needs to be complemented by other risk assessment methods (28). There are numerous risk assessment methods internationally, and Chinese scholars have conducted a lot of research on various OHRA methods (12, 29, 30). Nevertheless, it is still not clear what kinds of OHRA methods are currently being applied for occupational health risk assessment studies in China, what types of methodology studies on OHRA tools are conducted by Chinese researchers, and how applicable these methods are in OHRA in the workplaces. To further understand the progress of research on OHRA methodology in China, an aggregation and generalization of these OHRA methodological studies needs to be carried out. Therefore, we conducted this scope review to summarize the methodological researches on OHRA methods in China to provide information for future research on OHRA and occupational health risk management in China.

## Methods

### Study design

A scoping review was conducted to explore the research progress on OHRA methodology in China. The review proceeded five stages according to a scoping review method developed by Arksey and O'Malley (31), extended by Levac et al. (32), and further modified by Westphaln et al. (33). This scoping review provides an overview of the existing evidence on studies focus on OHRA methodology in China without a formal assessment of the methodological quality. The steps of the scoping review are: (1) identifying the research question; (2) identifying relevant literature; (3) selecting studies; (4) charting the data; and (5) collating, summarizing, and reporting the results. In order to enhance the quality of this scoping review, it was conducted and reported in accordance with the checklist of Preferred Reporting Items for Systematic Reviews and Meta-Analyses extension for Scoping Reviews (PRISMA-ScR) (34, 35).

### Research questions

Specific research questions of this scoping review were:

- What kinds of OHRA methods are currently involved in OHRA methodology studies in China?
- What types of studies have been conducted on these OHRA methods by Chinese researchers?
- What are the strengths, limitations and applicability of these OHRA methods?

### Search strategy

Focusing on OHRA methods, the authors systematically searched Chinese and English databases and relevant guideline websites from the date of establishment to June 30, 2022. Databases included Web of Science, PubMed, Scopus, the China National Knowledge Internet, WanFang Database. Search terms were developed based on three main concepts of “occupational health,” “risk assessment,” and “methods,” restricted to studies conducted in China and the language of literature were in Chinese or English. We selected synonyms, Medical Subject heading (MeSH) terms, and additional keywords and altered the final search string to match the syntax requirements of each database. The detailed search strategies for the respective databases were shown in Supplementary Table 1. Retrieved articles were initially reviewed by the title and the abstract to find potentially relevant studies and exclude irrelevant ones. Reference lists of relevant articles were reviewed to identify possible additional papers. We also searched additional web-based platforms such as Google and Baidu, as well as some government websites, university homepages and other websites in June 2022 to obtain relevant gray literature.

### Inclusion and exclusion criteria

Search results were screened in a reference manager by two reviewers (LZ and PX) to reduce bias and full-text screening was conducted only by the first author. Publications unrelated to the domain of this research were removed based on a review of their titles and abstracts. Unqualified records were excluded based on the exclusion criteria. The full text of article was retrieved and reviewed for more clarity if it was not satisfactorily removed based on the information available in title and abstract. Disagreements were resolved by including the articles in an in-depth analysis and discussion involved by the third reviewer (YZ).

Inclusion criteria of the study were as follows:

- Studies conducted with the working population in China.
- Methodological studies on OHRA methods.
- Studies published in English or Chinese.
- Peer-reviewed articles, gray literature (conference proceedings, thesis, government documents, and professional publications) explaining OHRA.

Exclusion criteria of the study were as follows:

- Literature for which full text was not available, if the key information we need was not available from the abstract.
- Letters to editors, editorials, short briefs, reviews, and study protocols.
- Literature that did not describe methodological issues on OHRA such as application, comparative, optimization, or modeling.
- Although the authors of the literature were Chinese, the workplaces studied were not in China.
- The focus of the article was outside the scope of this review.

### Data charting and analysis

Four researchers (FW, JZ, SW, and YH) were involved in data extraction and attended a training workshop focused on developing consistency across researchers by practicing the skills needed to reliable data extraction using a web-based form. To improve the accuracy of the literature information extracted, each researcher was randomly assigned to the same number of included publications, followed by an exchange review of the extracted information. Any disagreement was discussed and finalized by the four researchers to determine a unified opinion. A researcher (LZ) reviewed the extracted data for all the records included. The extracted information included year of publish, region of the institution of first author, type of study, OHRA tools involved, industries and types of hazardous if applicable, main results, strengths and limitations of OHRA methods, and main conclusions of the literature. A summary of the extracted data is available in Supplementary Table 2. The year of publication, the region of the researcher, the type of study, the type of hazard factors that each OHRA model can assess for the included literature were analyzed. The types of study included applied study, comparative study, and optimization study. Applied study is the practical application of occupational health risk assessment methods in one or more industries, with a description of the methodological characteristics. Comparative study focuses on the methodological principles, evaluation scope, strengths and limitations, and applicability of two or more OHRA methods to find the differences between the methods. Optimization study is conducted to optimize or improve one or more well-established OHRA methods and to compare the methodologies before and after the improvements.

## Results

**Figure 1:**
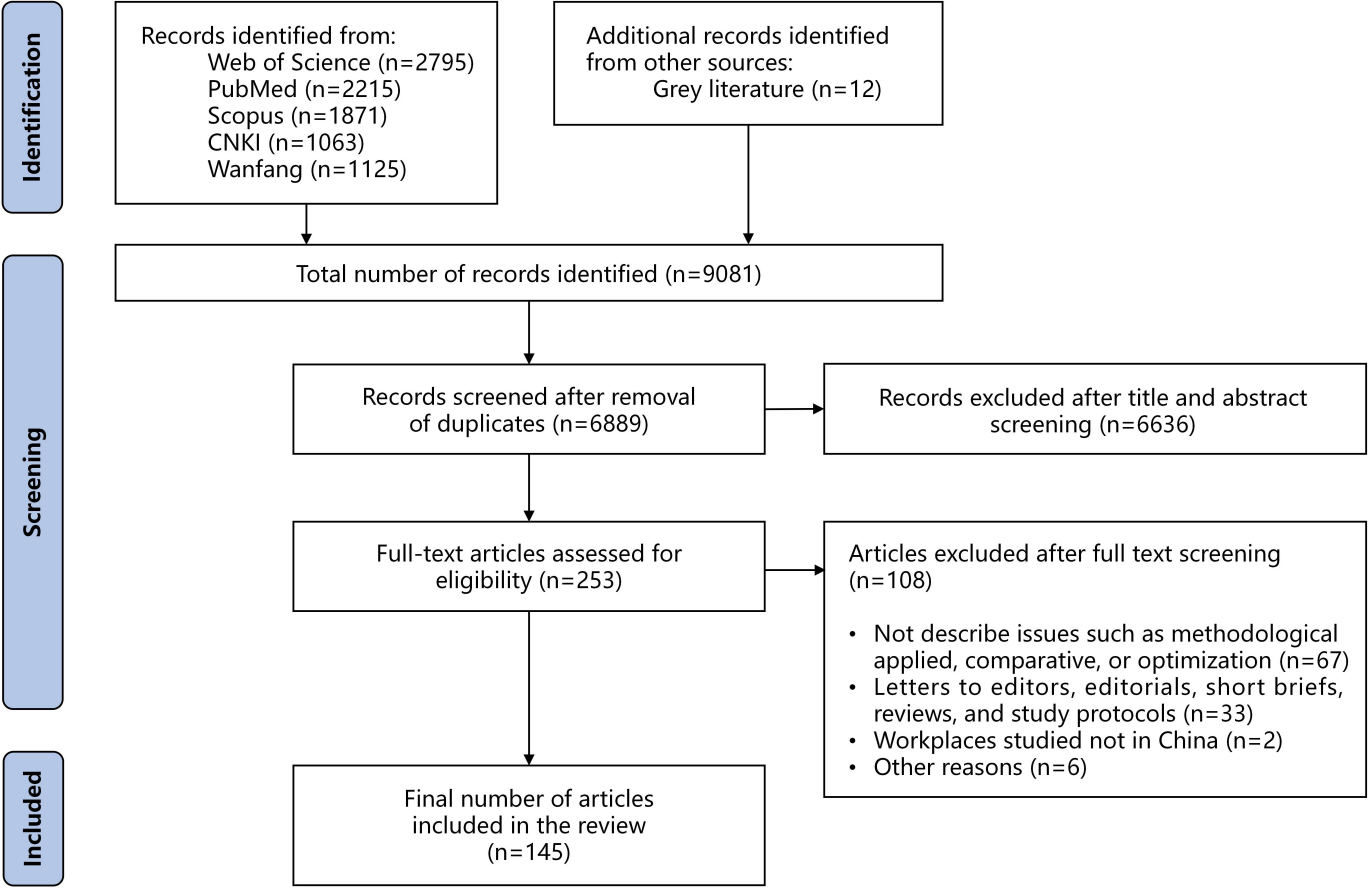
The PRISMA flowchart of this scoping review.

A total of 6,889 relevant non-duplicate records were identified from 9,081 records searched. After applying exclusion criteria, 253 articles were retrieved eligible for full-text screening, of which 145 records met inclusion criteria and were finally included for the review. The results of literature search by the two reviewers were generally consistent, except for disagreements on nine papers, which were resolved in discussion involved by the third reviewer. Figure 1 provides a summary of the PRISMA flowchart.

### Characteristics of studies included in this review

**Figure 2:**
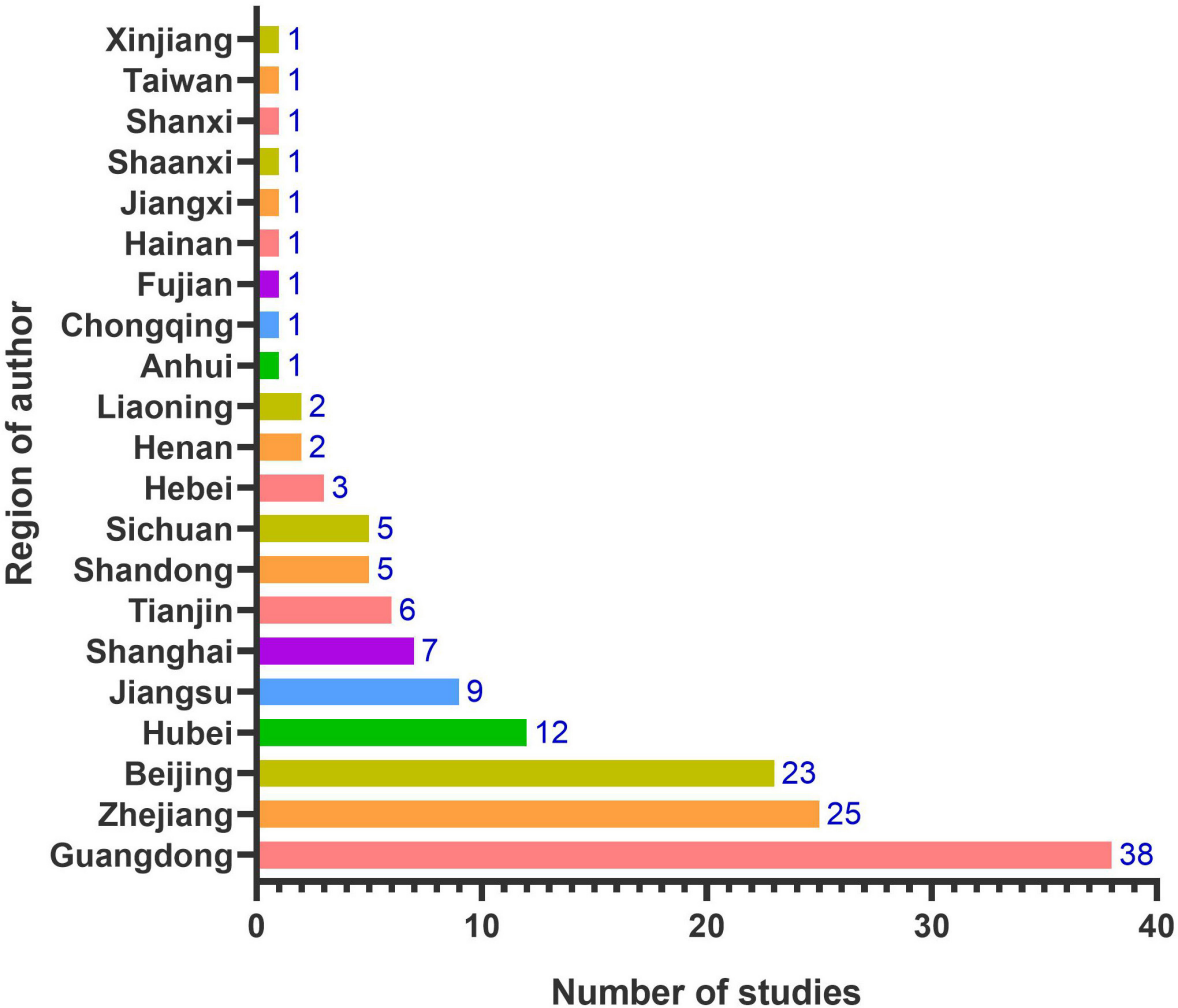
Region distribution of included studies based on the institutions of the first authors.

Table 1 shows that this review included 145 studies focusing on OHRA methodologies in China, most of which (*n* = 105, 72.4%) were published in 2018 and later. There were 108 applied studies, 30 comparative studies and seven optimization studies. There were 12 OHRA methods included in this review, including four qualitative methods, four quantitative methods, three semi-quantitative methods, and a comprehensive method. The most covered OHRA methods were the “Good Practice Guidance on OHRA” developed by the International Council on Mining and Metals (ICMM model), GBZ/T 298-2017, the “Semi-quantitative Method to Assess Occupational Exposure to Harmful Chemicals” (Singaporean model), the “Supplementary Guidelines for Inhalation Risk Assessment in Part F of the US Environmental Protection Agency's Risk Assessment Guidelines” (USEPA model), and the “Occupational Hazard Risk Index Evaluation Method” (OHR Index model). As shown in Figure 2, among the included literature, research institutions in Guangdong (*n* = 38, 26.2%) carried out the largest number of studies on OHRA, followed by Zhejiang (*n* = 25, 17.2%) and Beijing (*n* = 23, 15.9%).

**Table 1:** General characteristics of included studies.

	**Number**	**%**
**Year of publication**
Before 2018	40	27.6
In or after 2018	105	72.4
**Type of study**
Application study	108	74.5
Comparative study	30	20.7
Optimization study	7	4.8
**Classification of OHRA methods**
Qualitative	4	33.3
Quantitative	3	25.0
Semi-quantitative	4	33.3
Comprehensive	1	8.3
OHRA methods^*^
ICMM model	33	35.2
GBZ/T 298-2017	35	33.8
Singaporean model	30	31.0
USEPA model	24	22.1
OHR index model	16	15.2
Romanian model	7	11.0
Australian model	6	7.6
COSHH model	2	4.1
LEC model	4	4.1
Fuzzy model	4	3.4
Monte Carlo simulation	4	3.4
PBPK model	2	2.1

* Since a study may involve more than one OHRA method, the sum of the individual methods exceeds the total number of studies.

### Classification of OHRA methods in China

Over the decades, Chinese researchers have introduced some international risk assessment guidelines, from which have been applied and technically innovated in OHRA and promoted nationwide. Similar to the core principles of internationally used risk assessment models, most of the OHRA methods in China are based on hazard level, exposure level and probability of occurrence, and can be classified as qualitative, quantitative and semi-quantitative.

#### Qualitative OHRA methods

Qualitative occupational health risk assessment methods studied in China were mainly: (1) the “Risk Assessment Method for Occupational Accidents and Diseases” (Romanian model) developed by the Ministry of Labor and Social Protection in Romania (19); (2) the “Occupational Health and Safety Risk Assessment and Management Guideline” (Australian model) formulated by University of Queensland in Australia (18); (3) the ICMM model (36); and, (4) the “Control of Substances Hazardous to Health Essentials” (COSHH model) formulated by the United Kingdom Health and Safety Executive (17).

The Romanian model assesses the most severe consequences on the human body and probability of occurrence of risk factors in the workplaces, and determines the risk levels based on the combination of severity-likelihood levels (19). The Australian model uses a risk assessment calculator consisting of several connecting lines to determine the risk levels based on the likelihood of an outcome, the frequency of exposure and the severity of the outcome (18). The ICMM model comprehensively considers factors such as possible health consequences, exposure probability and exposure time, and determines the risk levels by the quantitative assignment method or matrix method. The quantitative assignment method of the ICMM model is used in the situation where the monitoring results of occupational disease hazards in the workplace do not exist, and the matrix method is used in the situation where the monitoring results exist (36). The COSHH model identifies the hazard level of chemicals according to the hazard term or occupational exposure limits (OEL), determines the exposure level according to the dustiness or volatility and usage, and then reaches the risk level and corresponding control measures according to the hazard level and exposure level (17).

#### Quantitative OHRA methods

In China, the most widely studied quantitative occupational health risk assessment method was the USEPA model (13). This risk assessment model can evaluate both of the carcinogenic and non-carcinogenic risks of a variety of chemicals with reference concentration (RfC) and inhalation unit risk (IUR) in the U.S. EPA website. Some Chinese researchers applied Monte Carlo simulation to OHRA as a complement to the USEPA model, especially in parametric uncertainty studies (37). Monte Carlo simulation is usually used to deal with the uncertainties associated with risk-related problems (38). It extrapolates population metrics based on sampling results to provide a quantitative approach to assessing the probability distribution of health risks. The Physiologically Based Pharmacokinetics (PBPK model) was also studied in China (39). The principle of the PBPK model is to construct a differential equation of mass conservation of chemical substances in the body, which requires the collection of various physiological parameters, partition coefficients, metabolic parameters, and absorption parameters. PBPK model was mainly used in researches such as chemical safety evaluation, drug metabolism analysis and new drug research and development, and are also used in health risk assessment of carcinogens (40).

#### Semi-quantitative OHRA methods

The “Semi-quantitative Method to Assess Occupational Exposure to Harmful Chemicals” (Singaporean model) established by the Ministry of Manpower of Singapore was the most widely used and researched semi-quantitative OHRA method in China (20). Other semi-quantitative OHRA methods studied in China included the fuzzy mathematical model (Fuzzy model), likelihood exposure consequence (LEC) model, and the “Occupational Hazard Risk Index Evaluation Method” (OHR Index model) (28, 41).

Risk levels in Singaporean model are calculated based on hazard ratings (HR), which is assigned based on the carcinogenicity classifications established by the American Conference of Governmental Industrial Hygienists (ACGIH) and the International Agency for Research on Cancer (IARC), and exposure ratings (ER), which is based on the ratio of the exposure level and OEL (20). The fuzzy mathematical model establishes a mathematical model according to the fuzzy mathematical membership theory: B = A × R. B is the total evaluation score, which is divided into excellent, good, general, poor, and very poor; A is the weight distribution set; R is a fuzzy matrix, which consists of monitoring values of occupational hazards (42). The LEC model uses the product of the index values from three factors related to occupational health to evaluate the health risk of workers (which refers to Danger, D). D = L × E × C, where D is the health risk; L is the possibility of the occurrence of the hazard; E is the frequency of the worker's exposure to the hazard; C is the possible consequence of the occurrence of the hazards (43, 44). The OHR Index model was established by Lin et al. (45) on the basis of the British occupational health and safety management system and the American occupational exposure assessment management strategy. The core principle of this method is that the risk index is the comprehensive calculation result of the health effect level, the exposure ratio and the operating condition level.

#### Chinese OHRA standard GBZ/T 298-2017

The GBZ/T 298-2017 is a comprehensive risk assessment guideline, including a qualitative model modified according to the COSHH model, a quantitative model modified according to the USEPA model, and semi-quantitative methods modified according to the Singapore model (25). The RfC and IUR values of some chemicals are provided in the quantitative assessment model in GBZ/T 298-2017. The semi-quantitative quantitative models in GBZ/T 298-2017 are exposure limit ratio method, exposure index method and comprehensive index method. The exposure limit ratio method and exposure index method basically follow the Singapore model. The exposure level of the comprehensive index method needs to consider the factors of chemical concentration in the air, physical and chemical properties, usage, exposure time and control measures (including engineering protection, personal protective equipment, emergency rescue measures and occupational health management) (28).

### Types of OHRA methodological studies in China

#### Applied studies

Applied research is mainly to apply one or more OHRA models to assess occupational health risks in one or more industries to find the applicability, strengths and limitations of the applied OHRA methods in specific industries. Researchers in China have used all of the above qualitative, quantitative and semi-quantitative OHRA methods to conduct applied studies on different types of occupational hazards in various industries, so as to explore the feasibility of applying these methods to occupational health risk assessment. For example, the results of applied research of Romanian model in precious metal smelter industry (46) and fluorescent lamp manufacturing industry (47) showed that although it is subjective and the possibility of consequences is not easy to determine, it could be used for OHRA of these industries. Huang et al. (48) applied the Singaporean model to assess the occupational health risk caused by chemicals in a dyestuff factory and found that this method is applicable and effective for OHRA.

#### Comparative studies

A comparative study compares the results of two or more OHRA models on occupational health risks in one or more industries. By qualitatively or quantitatively comparing the evaluation results of different methods, the differences in reliability and consistency between the methods as well as the strengths and limitations of methods can be drawn, which can provide a reference for the selection of OHRA methods (49). Xu et al. (30) compared the assessment results of six common occupational health risk assessment models (i.e., ICMM model, Singaporean model, USEPA Model, Romanian model, Australian model, and COSHH model) in leather, wooden furniture manufacturing, printing and dyeing, printing, and garment manufacturing industries. The results of this comparative study implied that the order of risk ratios (RR) between the six models was: EPA > COSHH > Singaporean > Australian > Romanian and ICMM; the USEPA model and Singaporean model had higher reliability; the USEPA model was relatively independent in methodology; the Singaporean model had the strongest correlation with other models; and combination of different methodologies could be a strategy for OHRAs. Tian et al. (29) conducted a comparative study on six types of OHRA models by expert consultation, literature summarization and key informant interviews, over-grading conversion and introduction of risk ratios to compare the consistency and correlation between the methods.

#### Optimization studies

The aim of an optimization study is to improve or optimize the commonly used risk assessment model, and use the optimized assessment model for occupational health risk assessment, and then evaluate the optimized model. The optimization studies carried by Luan et al. (50) and Gao et al. (51) provided ideas for the formulation of the semi-quantitative method in the GBZ/T 298-2017 in China, which considered the impact of engineering protection, personal protective equipment, emergency rescue and occupational health management on health risks compared with the Singaporean model (28). Luan et al. (50) added occupational health management and engineering control measures to improve the hazard level and exposure level evaluation of the Singaporean model and then applied the optimized model to the furniture manufacturing enterprises for occupational health assessment. The improved risk assessment model not only retained the strengths of the Singapore model, but also increased the risk assessment of physical factors. Zhang et al. (52) also built a new evaluation index based on four OHRA models to evaluate the risk of the hazards between industries.

### Strengths and limitations of OHRA methods

We extracted the assessment scope, strengths and limitations of all the OHRA methods included in this scoping review. The ICMM model, OHR Index model, Romanian model, Australian model, LEC model, and Fuzzy model can be applied to assess the occupational health risk caused by chemicals, physical factors, and dust. The Singaporean model, COSHH model, the GBZ/T 298-2017, Monte Carlo simulation, and PBPK model can be used to assess health risks from chemicals and dust, while USEPA Model can only assess the health risks caused by specific chemicals. Each method has its own strengths and limitations due to different evaluation principles, as shown in Table 2.

**Table 2:** Qualitative comparisons between OHRA models.

**Model**	**Classification**	**Scope**	**Strengths**	**Limitations**
ICMM model	Qualitative	Chemicals, physical factors, and dust	1. Broad scope 2. Application to various industries	1. Rely on subjective judgment 2. Has a possibility of overestimation
GBZ/T 298-2017	Comprehensive	Chemicals and dust	1. A combination of qualitative, quantitative and semi-quantitative methods 2. Suitable for different scenarios	1. Only considering exposure through inhalation 2. Cannot evaluate risks caused by physical factors
Singaporean model	Semi-quantitative	Chemicals and dust	1. Usage of exposure index method when air monitoring data are missing 2. High consistency with other methods	1. Relatively crude classification in terms of exposure index 2. Cannot evaluate risks caused by physical factors
USEPA Model	Quantitative	Chemicals	1. Quantitative assessment for the carcinogenic and non-carcinogenic risks 2. Scientific values of RfC and IUR based on epidemiological or toxicological data	1. Limited to chemicals with IUR and RfC values 2. No consideration for personal protective equipment 3. Difficult to differentiate multiple risk level
OHR Index model	Semi-quantitative	Chemicals, physical factors, and dust	Broad scope and easy to conduct	Rely on subjective judgment to get working condition grades
Romanian model	Qualitative	Chemicals, physical factors, and dust	1. Broad scope 2. Calculation of total risk level	1. Rely on subjective judgment 2. Difficult to judge the probability of a consequence occurring
Australian model	Qualitative	Chemicals, physical factors, and dust	1. Broad scope and easy to conduct 2. Appropriate for middle- and small-sized businesses	1. Rely on subjective judgment 2. Requirement of professional knowledge
COSHH model	Qualitative	Chemicals and dust	1. Simple and easy to conduct 2. Focus on middle- and small-sized businesses 3. To provide control measures	1. Overestimation of risk levels 2. Occurrence of bias when judging liquid volatility
LEC model	Semi-quantitative	Chemicals, physical factors, and dust	Broad scope and easy to conduct	Rely on subjective judgment
Fuzzy model	Semi-quantitative	Chemicals, physical factors, and dust	1. Has a wide range of application 2. Highly consistent with the evaluation results of the Singaporean model	Need data processing, not easy to conduct
Monte Carlo simulation	Quantitative	Chemicals and dust	Quantitative calculation, relatively objective	Not easy to conduct and limited scope
PBPK model	Quantitative	Chemicals and dust	Estimate internal exposure agent, relatively objective	Not easy to conduct and limited scope

## Discussion

Risk assessment of health risk is increasingly important to efficiently prevent and manage occupational diseases in the workplace. This scope review aimed to summarize the methodological studies on occupational health risk assessment methods in China. By searching major international and Chinese databases and relevant websites, we extracted 145 of the 9,081 searched papers that met the inclusion and exclusion criteria for this scoping review. Research on OHRA methodologies in China has increased significantly over the past 5 years, with most of the included studies published in 2018 and later. The regional distribution of first authors indicated that research institutions in Guangdong, Zhejiang and Beijing showed the highest interest in OHRA methodological research. The number of industrial enterprises and the level of economic development in these three regions are relatively developed within China (1).

The OHRA methods studied in the included literature were the ICMM model, GBZ/T 298-2017, the Singaporean model, the USEPA model, the OHR Index model, the Romanian model, the Australian model, the COSHH model, the LEC model, the Fuzzy model, the Monte Carlo simulation, and the PBPK model, ranked according to the number of studies. Researches on some of these methods has also been employed in other countries. American researchers have studied the USEPA model in the assessment of health risks of asbestos exposure and analyzed the strengths and limitations of the assess procedure (53). Golbabaei et al. (49) applied the Singaporean model to assess health risks of exposure to gases released by welding processes in natural gas transmission pipelines industry in Iran. In the U.S., Clewell et al. (54) described the process of the PBPK model development and highlighted issues related to the specification of model structure and parameters, model evaluation, and consideration of uncertainty in environmental and occupational risk assessment. Monte Carlo simulation was carried out to assess health risk of occupational exposure to heavy metals in a steel casting unit of a steelmaking plant in Iran (55).

OHRA methodological studies in China were mainly focused on applied studies, followed by comparative studies. The applied studies found that OHRA methods developed by different countries or international organizations had different principles and methodological characteristics (48, 56–63). Likewise, Mumtaz et al. (64) applied the PBPK model in some selected examples of environmental and occupational exposure assessments of chemicals and their mixtures to discuss the applicability of PBPK model in the U.S. The strengths, limitations and applicability of OHRA methods could be observed not only by carrying out applied studies, but also by conducting comparative studies (28–30, 65–67). Similar to the Chinese researchers, scholars in South Korea have also conducted a comparative study on a qualitative risk assessment method improved based on the COSHH model and a quantitative assessment improved based on USEPA model to evaluate health risks caused by 36 kinds of hazardous substances requiring management (68). In Iran, the results of a comparison study on health risk assessment on occupational exposure to styrene in a petrochemical industry using the USEPA model and the Singaporean model implied that the estimated health risk of exposure to styrene was higher in the EPA model than in the Singaporean model (69). Only a few studies included in this review were methodological optimization studies (45, 50–52, 70–72), which were conducted to improve the OHRA methods and provide insights for establishing OHRA methods suitable for the workplace in China. Optimization studies on OHRA methods have also been attempted in other countries. Ji et al. (73) in New Zealand revised the conventional risk assessment methods into a comprehensive risk assessment method with consideration of both safety accidents and chronic health issues, providing a way to include long-term health outcomes in OHRA.

The studied OHRA methods were divided into quantitative, semi-quantitative and qualitative methods, as well as a comprehensive method (i.e., the first OHRA guideline GBZ/T 298-2017 in China) that included a quantitative model, a qualitative model and three semi-quantitative models. Through this scoping review, we identified that various international and Chinese occupational health risk assessment methods have their own strengths, limitations and application scopes. The ICMM model, the OHR Index model, the Romanian model, the Australian model, the LEC model, and the Fuzzy model have the broadest range of assessments scope. These methods can be used to assess occupational health risks caused by nearly all kinds of hazards in various industries, though some of them may relatively rely on subjective judgment (42, 56, 63, 70, 74–76). Although the Singaporean model, the COSHH model, and the qualitative and semi-quantitative assessment models in GBZ/T 298-2017 cannot assess health risks caused by physical factors, they are simple and easy to operate, and are especially suitable for rapid assessment (26, 75, 77–79). The USEPA Model, the Monte Carlo simulation, and the PBPK model are objective methods, although the calculation processes are relatively complex, and the application scopes are limited (37, 39, 65, 80, 81).

Thus, there may not be a single model for a comprehensive risk assessment for all workplaces in all industries. Before applying them to OHRA in workplace, it is necessary to comprehensively consider the characteristics and evaluation principles of the methods and then choose a suitable OHRA method or combine multiple OHRA methods according to the characteristics of the workplaces (82, 83). Applicability of methodology is one of the most important issues that occupational health workers need to think deeply about. The Chinese occupational health risk evaluation standard GBZ/T 298-2017 has just been developed for 5 years and needs further improvement (27, 78, 84). Liang et al. (85) compared the results of four methods including GBZ/T 298-2017 to evaluate the risk of chemicals in the electrical appliance manufacturing industry. The result revealed that the quantitative method of GBZ/T 298-2017 may overestimate the health risk of chemicals. Tian et al. (78) carried out OHRA in battery manufacturing industries and indicated that the GBZ/T 298-2017 had several limitations, such as just considers exposure through inhalation route, cannot assess occupational health risks from physical factors, and the hazard classification of dust and chemical toxicants in semi-quantitative methods needs to be further refined. Therefore, it is necessary to strengthen the research on occupational health risk assessment methodology, and to establish and promote scientific, reasonable and operational occupational health risk assessment methods in line with China's national conditions in the future.

Using the established process outlined by Arksey and O'Malley (31) for conducting a scoping review, and reporting the results consistent with the PRISMA-ScR checklist, enhances the rigor and transparency of our review design, and trustworthiness of the results. We also anticipate that this review will provide insights for researchers focusing on OHRA methodological research. Probable limitations of this study must also be considered. Consistent with the limitations of the scope review, we did not systematically assess the methodological quality of the included studies in our review; however, this is a potential avenue for future systematic reviews and meta-analyses. Additionally, given the conceptual ambiguity regarding implementation outcome terminology (e.g., the multiple ways in which researchers define and discuss 'Applicable'), some literature that include OHRA methodological study may be excluded.

## Conclusion

The results of this scoping review indicated that occupational health risk assessment methodological research in China has been very popular in recent years. The most common OHRA methodological studies in China were applied studies, with some comparative studies and limited optimization studies. There are several types of OHRA methods studied, including qualitative, quantitative and semi-quantitative methods, as well as a comprehensive guideline proposed in China. Since each method has its strengths and limitations, the application of OHRA methods in occupational health risk assessment requires comprehensive consideration. At the same time, researches on the application of OHRA methods in more industries, quantitative comparative studies, optimization studies, and modeling studies of OHRA methods are essential to explore OHRA methods more suitable for workplaces in China.

## Data availability statement

The original contributions presented in the study are included in the article/Supplementary material, further inquiries can be directed to the corresponding authors.

## Author contributions

LZ and HZ designed this scoping review, search strategy, searched databases, and conducted data analysis and interpretation. LZ, PX, and YZ conducted the article screening process. FW, JZ, SW, and YH were involved in full-text reviewing of articles during the final stage of literature screening and extract information from literature. LZ and XL drafted the manuscript. All authors reviewed and approved it, contributed to the article, and approved the submitted version.

## Funding

This research was funded by the Zhejiang Provincial Key Research and Development Project (Grant Number: 2015C03039); the Zhejiang Provincial Program for the Cultivation of High-Level Innovative Health Talents, Zhejiang Province, China; and the Health Commission of Zhejiang Province (2019KY057).

## Conflict of interest

The authors declare that the research was conducted in the absence of any commercial or financial relationships that could be construed as a potential conflict of interest.

## Publisher's note

All claims expressed in this article are solely those of the authors and do not necessarily represent those of their affiliated organizations, or those of the publisher, the editors and the reviewers. Any product that may be evaluated in this article, or claim that may be made by its manufacturer, is not guaranteed or endorsed by the publisher.
