# Supplementary material for: Occupational health risk assessment methods in China: A scoping review
Source: Front Public Health. 2022 Nov 17;10:1035996. doi: 10.3389/fpubh.2022.1035996 (PMC9714297; doi:10.3389/fpubh.2022.1035996)
Supplement: Supplementary file 1 [file Data_Sheet_1.docx]

**Supplementary Material**

1. **Table S1: Search strategies employed in the scoping review**

| **Databases** | **Strategy** | **Results** |
| --- | --- | --- |
| Web of Science- 1900 to June 30, 2022 | (((TS=(occupational health) OR TS=(occupational*)) AND (TS=(risk) OR TS=(hazard)) AND (TS=(assessment) OR TS=(evaluation)) AND (TS=(method*) OR TS=(tool*) OR TS=(model*))) OR ((TI=(occupational health) OR TI=(occupational*)) AND (TI=(risk) OR TI=(hazard)) AND (TI=(assessment) OR TI=(evaluation)) AND (TI=(method*) OR TI=(tool*) OR TI=(model*)))) AND (TS=China OR AD=China) | 2795 |
| PubMed- 1946 to June 30, 2022 | ((("Risk Assessment"[Mesh]) AND "Occupational Health"[Mesh]) OR ((occupational* OR occupational health) AND (risk[Title/Abstract] OR hazard[Title/Abstract]) AND (assessment OR evaluation) AND (method* OR tool* OR model*))) AND (China) | 2215 |
| Scopus- 1788 to June 30, 2022 | ( TITLE-ABS-KEY ( occupational AND health ) OR TITLE-ABS-KEY ( occupational* ) ) AND ( TITLE-ABS-KEY ( risk ) OR TITLE-ABS-KEY ( hazard ) ) AND ( TITLE-ABS-KEY ( assessment ) OR TITLE-ABS-KEY ( evaluation ) ) AND ( ALL ( method* ) OR ALL ( tool* ) OR ALL ( model* ) ) AND ( TITLE-ABS-KEY ( China ) OR AFFILCOUNTRY ( China ) ) | 1871 |
| The China National Knowledge Internet- 1915 to June 30, 2022 ^*^ | ( ( ( 主题%= 风险评估+风险评价+危险度评估+危险性评估+危险度评价+危险性评价 or 题名%= 风险评估+风险评价+危险度评估+危险性评估+危险度评价+危险性评价 ) AND ( 主题%= 职业健康+职业病危害+职业危害+职业暴露健康 or 题名%= 职业健康+职业病危害+职业危害+职业暴露健康 ) ) AND ( 主题%= 模型+方法+工具 or 题名%= 模型+方法+工具 ) ) | 1063 |
| WanFang Database- 1900 to June 30, 2022 ^*^ | (主题:(("风险评估" or "风险评价" or "危险度评估" or "危险性评估" or "危险度评价" or "危险性评价") and ("职业健康" or "职业病危害" or "职业危害" or "职业暴露健康") and ("模型" or "方法" or "工具"))) | 1125 |
| **Total Results** |  | 9069 |

^*^Chinese words corresponding to English words:主题: topic; 题名: title; 风险/危险度/危险性: risk; 评价/评估: assessment/evaluation; 职业健康/职业暴露健康: occupational health; 职业危害/职业病危害: occupational hazard; 模型: model; 方法: method; 工具: tool.

1. **Table S2: Characteristics of included studies**

| **Title** | **Year of publication** | **Reference type** | **Author** | **Region** | **Study type** | **OHRA methods** | **Industries** | **Hazards** | **Language** |
| --- | --- | --- | --- | --- | --- | --- | --- | --- | --- |
| A preliminary study on the evaluation method of occupational hazard risk index (1) | 2006 | Journal Article | Sihao Lin | Fujian | Optimization study | OHR Index model, LEC model | Shoemaking industry, automobile manufacturing industry | Chemical agents such as benzene, toluene, noise, dust | Chinese |
| Health risk assessment of occupational exposure to toxic substances (2) | 2007 | Journal Article | Deyin Huang | Tianjin | Applied study | Singaporean model | Chemical industry | Chemical agents such as bromine, bisphenol A, hydrogen peroxide, chlorobenzene | Chinese |
| Application of a health risk classification method to assessing occupational hazard in China (3) | 2009 | Conference Proceedings | Deyin Huang | Tianjin | Applied study | Singaporean model | Dyestuff manufacturing | Chemical agent such as aminobenzene and nitrobenzene, sulfur trioxide, sodium hydroxide | English |
| Research on risk assessment based on Monte Carlo simulation and dose-response multistage model (4) | 2010 | Conference Proceedings | Deyin Huang | Tianjin | Comparative study | PBPK model, Monte Carlo simulation | / | / | English |
| Application of LEC evaluation method in risk assessment of occupational disease hazards in construction projects (5) | 2011 | Journal Article | Qin Ji | Jiangsu | Applied study | LEC model | Chemical industry | Chemical agents such as methanol, nitrogen oxides, carbon monoxide and carbon dioxide | Chinese |
| Research on the occupational hazards risk assessment in coal mine based on the hazard theory (6) | 2011 | Journal Article | Zhu-Wu Zhu | Shandong | Applied study | Fuzzy model | Coal mining industry | Chemical agents: nitrogen oxides, carbon oxide, H_2_S, noise, dust | English |
| Assessment of noise and heavy metals (Cr, Cu, Cd, Pb) in the ambience of the production line for recycling waste printed circuit boards (7) | 2012 | Journal Article | Mianqiang Xue | Shanghai | Applied study | USEPA Model | Waste printed circuit boards (PCBs) recycling industry | Heavy metals (Cr, Cu, Cd, Pb) and noise | English |
| Application of risk assessment method for occupational hazard in dye chemical enterprise (8) | 2013 | Journal Article | Caixia Jiang | Zhejiang | Applied study | Singaporean model | Chemical industry | Chemical agents such as nitrochlorobenzene, sulfuric acid, nitric acid | Chinese |
| An application study of Australian Occupational Risk Assessment Model in a certain battery production enterprise (9) | 2013 | Journal Article | Shasha Wang | Zhejiang | Applied study | Australian model | Battery production industry | Lead fume, lead dust, chemical agents such as sulfuric acid, physical agents such as noise and high temperature | Chinese |
| Occupational disease hazards evaluation in an enterprise by a Semi-Quantitative risk assessment method (10) | 2013 | Journal Article | Zhiping Wang | Zhejiang | Applied study | Singaporean model | Battery production industry | Lead dust, lead fume, sulfuric acid | Chinese |
| Application of U.S. EPA inhalation risk model to occupational health risk assessment of wooden furniture manufacturing factories (11) | 2014 | Journal Article | Bopeng Leng | Zhejiang | Applied study | USEPA Model | Wooden furniture manufacturing industry | Chemical agents such as benzene, toluene, xylene, ethyl acetate, physical agents such as noise, dust | Chinese |
| Application of three risk assessment methods on chemicals risk assessment and management (12) | 2014 | Journal Article | Cuiju Wen | Guangdong | Applied study | Singaporean model, OHR Index model | Wastewater treatment industry | Chemical agents such as benzene, toluene, xylene, non-methane total hydrocarbons, solvent gasoline, hydrogen sulfide | Chinese |
| A study on application of inhalation risk assessment model of USEPA in occupational health risk assessment (13) | 2014 | Journal Article | Lifang Zhou | Zhejiang | Applied study | USEPA Model | Papermaking industry, chemical industry, electroplating industry | Chemical agents such as hydrogen sulfide, methanol, hydrogen chloride, methyl chloride, sulfuric acid, sodium hydroxide, physical agents such as noise and high temperature, dust | Chinese |
| PM2.5, PM10 and health risk assessment of heavy metals in a typical printed circuit boards manufacturing workshop (14) | 2014 | Journal Article | Peng Zhou | Shanghai | Applied study | USEPA Model | Waste printed circuit boards (PCBs) recycling industry | PM2.5, PM10 and heavy metals | English |
| Health risk of Shenzhen gas station workers exposed to MTBE: a primary study based on PBPK model (15) | 2014 | Journal Article | Siyang Ye | Guangdong | Applied study | PBPK model | Petrol station | Methyl tert-butyl ether (MTBE) | Chinese |
| Application of LEC method in occupational health risk assessment of ceramic enterprise (16) | 2014 | Journal Article | Songgen Chen | Guangdong | Applied study | LEC model | Ceramic manufacturing industry | Chemical agents such as CO, CO_2_, physical agents such as noise, high temperature, dust | Chinese |
| A study on application of five risk assessment methods for occupational health (17) | 2014 | Thesis | Xiaoyan Li | Zhejiang | Applied study | ICMM model, Singaporean model, USEPA Model, Romanian model, Australian model | Thermal power generation industry | Coal dust, silica dust, physical agents such as noise, industrial frequency electric field, chemical agents such as ammonia, carbon monoxide, sulfur dioxide, nitrogen dioxide, nitric oxide | Chinese |
| Comparative study on occupational risk assessment using two foreign models (18) | 2015 | Journal Article | Weiming Yuan | Zhejiang | Comparative study | Singaporean model, USEPA Model | Furniture manufacturing industry | Chemical agents such as benzene, toluene, xylene, formaldehyde, physical agents such as noise, dust | Chinese |
| Health risk assessment of toxic VOCs species for the coal fire well drillers (19) | 2015 | Journal Article | Yulong Yan | Shanxi | Applied study | USEPA Model, Monte Carlo simulation | Coal mining industry | Volatile organic compounds | English |
| Health risk assessment for inhalation exposure to methyl tertiary butyl ether at petrol stations in southern China (20) | 2016 | Journal Article | Dalin Hu | Guangdong | Applied study | USEPA Model | Petrol station | Methyl tertiary butyl ether (MTBE) | English |
| A Study on application and modification of three occupational health risk assessment models (21) | 2016 | Thesis | He Gao | Xinjiang | Optimization study | Singaporean model, USEPA Model, Romanian model | Furniture manufacturing industry | Chemical agents such as formaldehyde, benzene, toluene, xylene, dust | Chinese |
| Application of semi-quantitative risk assessment model in glyphosate industry in Singapore (22) | 2016 | Journal Article | Hong Fu | Zhejiang | Applied study | Singaporean model | Glyphosate manufacturing industry | Chemical agents such as methanol, triethylamine, methyl chloride | Chinese |
| Application of two risk assessment models to printing industry (23) | 2016 | Journal Article | Hongwei Xie | Zhejiang | Applied study | ICMM model, Singaporean model | Printing industry | Chemical agents such as benzene, toluene, xylene, acetone | Chinese |
| ICMM risk assessment technology－based research on quantitive and qualitative assessment of a electroplating enterprise on occupational health risks (24) | 2016 | Journal Article | Pengbo Leng | Zhejiang | Applied study | ICMM model | Electroplating industry | Chemical agents such as sodium hydroxide, hydrochloric acid, sulfuric acid, chromate | Chinese |
| Application of ICMM occupational health risk assessment model in evaluation of occupational risk of a refractory enterprise (25) | 2016 | Journal Article | Qingxia Min | Liaoning | Applied study | ICMM model | Refractory material manufacturing industry | Dust, physical agents such as noise, high temperature, chemical agents such as carbon monoxide | Chinese |
| Romanian method for risk assessment of occupational accidents and diseases application effect in a precious metal smelter (26) | 2016 | Journal Article | Xiaofeng Yu | Zhejiang | Applied study | Romanian model | Metal smelting industry | Lead dust, lead fume, physical agents such as noise, high temperature, chemical agents such as sulfuric acid, hydrochloric acid, sulfur dioxide, ammonia, hydrazine, sodium carbonate, nitric oxide, nitrogen dioxide | Chinese |
| Application of LEC method in occupation health risk assessment of toner production enterprise (27) | 2016 | Journal Article | Xiaoou Tang | Guangdong | Applied study | LEC model | Printing consumables manufacturing industry | Dust, physical agents such as noise, chemical agents such as styrene, butyl acrylate | Chinese |
| Application of Graham method in health risk assessment of occupational hazards (28) | 2016 | Journal Article | Yinan Cao | Liaoning | Applied study | LEC model | Thermal power generation industry | Chemical agents such as ammonia, carbon monoxide, sulfur dioxide, nitrogen monoxide, nitrogen dioxide, physical agents such as high temperature | Chinese |
| Application of three risk assessment models in occupational health risk assessment of dimethylformamide (29) | 2016 | Journal Article | Zhijun Wu | Beijing | Applied study | Singaporean model, USEPA Model | DMF manufacturing industry | Dimethylformamide | Chinese |
| Application of two risk assessment methods to occupational health risk assessment in a ferrous metal foundry (30) | 2017 | Journal Article | Aihong Wang | Zhejiang | Applied study | ICMM model, OHR Index model | Ferrous metal foundry industry | Dust | Chinese |
| Study on application of two risk assessment methods in coal dust occupational health risk assessment (31) | 2017 | Journal Article | Bin Wu | Beijing | Applied study | OHR Index model | Coal mining industry | Dust | Chinese |
| Application of Semi-quantitative Risk Assessment Technology in Electronic Enterprises (32) | 2017 | Journal Article | Guangyuan Huang | Beijing | Applied study | Singaporean model | Electronics manufacturing industry | Benzene, toluene, xylene, formaldehyde | Chinese |
| A comparative study on the application of different methods of occupation health risk assessment in small furniture manufacturing industry (33) | 2017 | Journal Article | Guolin Bian | Jiangsu | Applied study | Singaporean model, USEPA Model, Australian model | Wooden furniture manufacturing industry | Wood dust, physical agents such as noise, chemical agents such as formaldehyde and benzene | Chinese |
| Health risk assessment method for Chinese stone quarrying based on ICMM evaluation method (34) | 2017 | Journal Article | Jingdong Zhang | Hubei | Applied study | ICMM model | Stone quarrying industry | Dust, noise | Chinese |
| Application of EPA inhalation risk assessment model in mercury occupational health risk in fluorescent lamp manufacturing enterprises (35) | 2017 | Journal Article | Lifang Han | Zhejiang | Applied study | USEPA Model | Fluorescent lamp manufacturing industry | Dust, physical agents such as noise, high temperature, chemical agents such as carbon monoxide, carbon dioxide | Chinese |
| Application of two health risk assessment models in the occupational health risk assessment of chemicals in different industries (36) | 2017 | Journal Article | Lifang Zhou | Zhejiang | Applied study | Singaporean model, Romanian model | Papermaking industry, chemical industry, electroplating industry | Dust, chemical agents such as methanol, xylene, sodium hydroxide, hydrogen chloride, hydrogen cyanide, sulfuric acid and chromates | Chinese |
| A study on the application of Romania risk assessment method of occupational accidents and diseases in a certain fluorescent lamp manufacture enterprise (37) | 2017 | Journal Article | Ming Li | Zhejiang | Applied study | Romanian model | Fluorescent Lamp Manufacturing Industry | Dust, physical agents such as noise and high temperature, chemical agents such as carbon monoxide, carbon dioxide | Chinese |
| Comparing the risk of formaldehyde in a plywood manufacturing factory with two risk assessment methods (38) | 2017 | Journal Article | Qingrong Wang | Shandong | Applied study | Singaporean model, USEPA Model | Plywood manufacturing industry | Formaldehyde | Chinese |
| Occupational semi-quantitative risk assessment in a crane manufacturing enterprise (39) | 2017 | Journal Article | Tao Liu | Zhejiang | Applied study | Singaporean model | Crane manufacturing industry | Chemical agents such as benzene, toluene, xylene, manganese and its compounds, butanol, butyl acetate, physical agents such as noise, welding fumes, silica dust, grinding wheel dust | Chinese |
| A study on application of semi-quantitative risk assessment models in furniture industry (40) | 2017 | Journal Article | Yuqing Luan | Zhejiang | Optimization study | Singaporean model | Furniture manufacturing industry | Wood dust, physical agents such as noise, chemical agents such as formaldehyde, benzene, toluene and xylene | Chinese |
| Occupational health risk assessment of benzene exposure among enterprises (41) | 2018 | Journal Article | Bo Wu | Beijing | Applied study | Singaporean model, OHR Index model | Furniture manufacturing industry, transformer manufacturing industry | Benzene | Chinese |
| Assessment on occupational health risks of 1,3-butadiene exposure by two risk assessment models (42) | 2018 | Journal Article | Bo Wu | Beijing | Applied study | Singaporean model, OHR Index model | Rubber production industry | 1,3-Butadiene | Chinese |
| Qualitative and quantitative differences between common occupational health risk assessment models in typical industries (43) | 2018 | Journal Article | Fang Tian | Shanghai | Comparative study | ICMM model, Singaporean model, USEPA Model, Romanian model, Australian model, COSHH model | Wooden furniture manufacturing industry, electroplating industry, crane manufacturing industry | Chemical agents, dust, physical agents | English |
| Risk assessment of pneumoconiosis in the ceramic production and refractory manufacturing (44) | 2018 | Journal Article | Fei Ye | Beijing | Applied study | ICMM model, GBZ/T 298-2017 | Ceramic manufacturing industry, refractory material manufacturing industry | Dust | Chinese |
| Occupational health risk assessment of coal industry chain (45) | 2018 | Journal Article | Feng Han | Beijing | Applied study | ICMM model, OHR Index model | Coal mining industry | silica | Chinese |
| Comparative study on application of different occupational health risk assessment methods in lead-acid battery production industry (46) | 2018 | Journal Article | Hongying Bian | Beijing | Comparative study | GBZ/T 298-2017 | Lead-acid battery industry | Lead fume, lead dust, sulfuric acid | Chinese |
| Comparison of occupational health risk assessment of an electronic enterprise based on EPA method and occupational disease work classification method (47) | 2018 | Journal Article | Jingdong Zhang | Hubei | Comparative study | Singaporean model, USEPA Model | Electronics manufacturing industry | Dust; chemical agents such as nitrogen dioxide, sulfuric acid, xylene; physical agents such as noise | Chinese |
| Comparison of application effects of several occupational health risk assessment methods in quarrying industry (48) | 2018 | Journal Article | Jingdong Zhang | Hubei | Comparative study | ICMM model, Romanian model | Stone quarrying industry | Dust, noise | Chinese |
| Application of ICMM occupational Health Risk Assessment Modelin evaluation of occupational risk of a lead-acid battery enterprise (49) | 2018 | Journal Article | Lin Chen | Jiangsu | Applied study | ICMM model | Lead-acid battery industry | Lead fume, lead dust, sulfuric acid | Chinese |
| Occupational hazards in a lead—acid battery enterprise：a comparison study of three health risk assessment methods (50) | 2018 | Journal Article | Lin Chen | Jiangsu | Applied study | ICMM model, GBZ/T 298-2017 | Lead-acid battery industry | Lead fume, lead dust, sulfuric acid | Chinese |
| Application of Singapore occupational hazards semi—quantitative risk assessment method in a lead—acid battery enterprise (51) | 2018 | Journal Article | Lin Chen | Jiangsu | Applied study | Singaporean model | Lead-acid battery industry | Lead fume, lead dust, sulfuric acid | Chinese |
| Application of three risk assessment models in occupational health risk assessment of toluene (52) | 2018 | Journal Article | Man Zhang | Beijing | Applied study | Singaporean model, USEPA Model | Tape production and use industry, automobile parts manufacturing, printing industry, pharmaceutical material production industry | Toluene | Chinese |
| Assessment of the inhalation risks associated with working in printing rooms: a study on the staff of eight printing rooms in Beijing, China (53) | 2018 | Journal Article | Mingxing Su | Beijing | Applied study | USEPA Model | Printing industry | Formaldehyde, benzene, toluene, xylene, and PM2.5 | English |
| Comparison of two methods of evaluating occupational health risk in a shoemaking enterprise (54) | 2018 | Journal Article | Peixian Chen | Guangdong | Comparative study | Singaporean model | Shoemaking industry | Chemical agents such as xylene, ethyl acetate, butyl acetate, n-hexane, 1,2-dichloroethane | Chinese |
| Applied study and case report of comparison and application of two occupational health risk assessment methods in chair furniture manufacturing enterprises (55) | 2018 | Journal Article | Peng Zhang | Zhejiang | Comparative study | ICMM model, Singaporean model | Furniture manufacturing industry | Wood dust; chemical agents such as aldehydes, benzene, toluene, xylene, dichloromethane, dichloroethane, ethyl acetate; physical agents such as noise. | Chinese |
| Occupational health risk assessment for workers exposed to low concentration of benzene (56) | 2018 | Journal Article | Pengbo Leng | Zhejiang | Applied study | USEPA Model | Manufacturing industry | Benzene | Chinese |
| Application of two risk assessment methods on occupational health risk assessment of wet sand casting molding process (57) | 2018 | Journal Article | Shihe Ding | Anhui | Applied study | ICMM model | Foundry industry | Chemical agents such as formaldehyde, phenol (phenol, cresol), triethylamine, methanol, physical agents such as noise, silica dust | Chinese |
| Application of two occupational health risk assessment models in an automobile-component manufactory in Fengxian District of Shanghai (58) | 2018 | Journal Article | Suhong Cao | Shanghai | Applied study | Singaporean model, Fuzzy model | Automotive parts manufacturing industry | Chemical agents such as toluene, formaldehyde, copper fume, dust | Chinese |
| Application of occupational health risk assessment model of International of Council on mining and metals in calcium carbonate production enterprises (59) | 2018 | Journal Article | Tusen Huang | Guangdong | Applied study | ICMM model | Calcium carbonate production industry | Physical agents such as noise; limestone dust, welding fumes; chemical agents such as hydrogen chloride and hydrochloric acid. | Chinese |
| Application research of three risk assessment methods to organic solvents in painting produced industry (60) | 2018 | Journal Article | Xudong Li | Guangdong | Applied study | ICMM model, GBZ/T 298-2017 | Paint production industry | Benzene, toluene, xylene, styrene, ethyl acetate, butyl acetate, toluene diisocyanate, methyl methacrylate, isopropanol, n-butanol and acetone. | Chinese |
| Application of three models for occupational health risk assessment to a transformer factory in Shenzhen City (61) | 2018 | Journal Article | Yafeng Tian | Guangdong | Applied study | GBZ/T 298-2017 | Transformer manufacturing industry | Lead fumes, chemical agents such as benzene, toluene, xylene, isopropyl alcohol | Chinese |
| Comparison of the application of three occupational health risk assessment models in battery manufacturers (62) | 2018 | Journal Article | Yafeng Tian | Guangdong | Applied study | GBZ/T 298-2017 | Lead-acid battery industry | Lead dust, lead fume; sulfuric acid, sodium hydroxide, butyl acetate | Chinese |
| Comparison of qualitative and semi-quantitative occupational health risk assessment methods in an adhesive manufacturer (63) | 2018 | Journal Article | Yaling Zou | Guangdong | Comparative study | ICMM model, Singaporean model, Romanian model | Adhesive production industry | Toluene, acetone, butanone, methyl acetate, ethyl acetate, butyl acetate, tetrachloroethylene, cyclohexane, n-heptane, n-hexane, methyl methacrylate and dichloromethane | Chinese |
| Occupational health risk assessment in the electronics industry in China based on the occupational classification method and EPA model (64) | 2018 | Journal Article | Ying Cai | Hubei | Comparative study | USEPA Model | Electronics industry | Chemical agents such as ammonia, chlorine, ozone, fluoride, sulfuric acid, hydrogen chloride, ethylene glycol, phosphine, boron trifluoride, isopropanol, and MACHs. | English |
| Comparison of multiple risk assessment methods for occupational health risk assessment for aluminum dust post (65) | 2018 | Journal Article | Zhiming Liang | Guangdong | Comparative study | ICMM model, GBZ/T 298-2017, LEC model | Automobile parts manufacturing industry, appliance manufacturing industry，construction material production industry | Aluminum dust | Chinese |
| Occupational health risk assessment of 1,2-dichloroethane for toy manufacturing enterprises in Shantou (66) | 2018 | Journal Article | Zixu Lin | Guangdong | Applied study | OHR Index model | Toy manufacturing industry | 1,2-Dichloroethane | Chinese |
| Occupational health risk assessment of low concentrations benzene toluene and xylenes (67) | 2019 | Journal Article | Aihong Wang | Zhejiang | Applied study | GBZ/T 298-2017 | Wooden furniture manufacturing industry, shoemaking industry, automobile parts manufacturing industry | Benzene, toluene, xylene | Chinese |
| ICMM risk assessment model assessing a coal-fired power plants’ occupational health risk (68) | 2019 | Journal Article | Bin Liu | Henan | Applied study | ICMM model | Thermal power generation industry | Coal dust, silica dust; physical agents such as noise; chemical elements such as ammonia, sulfide, barium hydroxide | Chinese |
| Improvement and application research on evaluation method of coal mine dust hazard degree (69) | 2019 | Thesis | Donghua Wang | Beijing | Applied study | ICMM model, GBZ/T 298-2017, USEPA Model, Romanian model, Australian model | Coal mining industry, power generation industry, coal chemical industry | Dust | Chinese |
| Application of two risk assessment methods in occupational health risk of 2-butoxyethonal (70) | 2019 | Journal Article | Haibin Li | Beijing | Applied study | Singaporean model | Bicycle manufacturing industry, printing industry, automobile manufacturing | 2-Butoxyethanol | Chinese |
| Comparative study of three semi-quantitative risk assessment methods in risk classification of silica dusts exposed posts (71) | 2019 | Journal Article | Hongying Bian | Beijing | Comparative study | ICMM model, GBZ/T 298-2017 | Mining industry, construction industry | Dust | Chinese |
| A comparative study of three risk assessment methods on evaluation of risk rating for dust hazard in an offshore oil engineering equipment manufacturing project of Guangdong Province (72) | 2019 | Journal Article | Huifeng Chen | Guangdong | Comparative study | ICMM model, OHR Index model | Offshore petroleum engineering equipment manufacturing industry | Dust such as welding fumes, grinding wheel grinding dust; physical agents such as noise | Chinese |
| Application research of four occupational health risk assessment methods in chemical enterprises (73) | 2019 | Thesis | Jun Ding | Guangdong | Applied study | ICMM model, GBZ/T 298-2017 | Chemical Industry | n-hexane, cyclohexane, benzene, toluene, xylene, ethyl acetate, methyl methacrylate, butyl methacrylate, styrene, alcohol methyl isobutyl ketone, acetic acid, n-butanol | Chinese |
| Comparison of three health risk assessment methods for organic solvents in furniture manufacturing enterprises (74) | 2019 | Journal Article | Jun Ding | Guangdong | Comparative study | GBZ/T 298-2017 | Furniture manufacturing industry | Benzene, toluene, xylene, ethyl acetate, n-hexane, cyclohexane, dichloromethane and styrene | Chinese |
| Application of 3 methods on occupational health risk assessment of methyl isoamyl ketone (75) | 2019 | Journal Article | Lei Wang | Shandong | Applied study | OHR Index model, Romanian model | Chemical industry | Methyl isoamyl ketone | Chinese |
| Application of three methods in occupational health risk assessment of automobile 4S shop (76) | 2019 | Journal Article | Lin Chen | Guangdong | Applied study | ICMM model, GBZ/T 298-2017 | Automobile sales and repair service industry | Dust; chemical agents such as benzene, toluene, xylene, ethylbenzene, ethyl acetate, butyl acetate, acetone, n-hexane, n-butanol; physical agents such as noise | Chinese |
| Quantitative health risk assessment of inhalation exposure to automobile foundry dust (77) | 2019 | Journal Article | Ruipeng Tong | Beijing | Applied study | USEPA Model, Monte Carlo simulation | Automobile manufacturing industry | Dust | English |
| Application of ICMM occupation health risk assessment model in occupational hazard assessment of a coking plant in Chengde City (78) | 2019 | Journal Article | Shuqing Zou | Hebei | Applied study | ICMM model | Coal processing industry | Dust; physical agents such as noise, high temperature; chemical agents such as naphthalene, phenol, hydrogen cyanide, carbon monoxide, sulfur dioxide, benzene and benzene homologues, ammonia, hydrogen sulfide | Chinese |
| Application of Fuzzy Mathematics Method in risk assessment of occupational disease hazards in wooden furniture manufacturing enterprises (79) | 2019 | Journal Article | Tusen Huang | Guangdong | Applied study | Fuzzy model | Wooden furniture manufacturing industry | Wood dust; physical agents such as noise, vibration; chemical agents such as benzene, toluene, xylene, formaldehyde and ethyl acetate | Chinese |
| Application of three semi-quantitative risk assessment methods in electroplating enterprises (80) | 2019 | Journal Article | Weilan Yan | Guangdong | Applied study | GBZ/T 298-2017 | Electroplating industry | Chemical agents such as sulfuric acid, sodium hydroxide, nickel sulfate, hydrogen cyanide, butanol, ethyl acetate, benzene, toluene, xylene | Chinese |
| Application of Singapore semi-quantitative risk assessment model for occupational exposure to chemical toxicants in automotive manufacturing industry (81) | 2019 | Journal Article | Xin Li | Chongqing | Applied study | Singaporean model | Automobile manufacturing industry | Welding fumes; chemical agents such as nitrogen dioxide, carbon monoxide, manganese and its compounds, cyclohexanone | Chinese |
| Application of occupational health risk assessment model in glyphosate production enterprises (82) | 2019 | Journal Article | Xin Liu | Jiangsu | Applied study | ICMM model, Singaporean model | Glyphosate manufacturing industry | Glyphosate | Chinese |
| Application of semi-quantitative risk assessment method in occupational health risk assessment of wooden furniture manufacturing enterprises (83) | 2019 | Journal Article | Yan Yang | Guangdong | Applied study | GBZ/T 298-2017 | Wooden furniture manufacturing industry | Dust; chemical agents such as formaldehyde, toluene, xylene, ethyl acetate, butyl acetate, n-hexane | Chinese |
| Application of occupational health risk assessment method in dioctyl phthalate producing enterprise (84) | 2020 | Journal Article | Chengming Meng | Henan | Applied study | GBZ/T 298-2017 | Chemical industry | DEHP | Chinese |
| Application of occupational health risk assessment method in lead zinc mining industry (85) | 2020 | Journal Article | Hui Wang | Sichuan | Applied study | GBZ/T 298-2017 | Lead and zinc mining industry | Silica dust, lead dust | Chinese |
| Occupational health risk assessment of a lead -acid battery enterprise based on two models of ICMM occupational health risk assessment (86) | 2020 | Journal Article | Jianguo Lu | Guangdong | Applied study | ICMM model | Lead-acid battery industry | Chemical agents such as lead, sulfuric acid; physical agents such as noise | Chinese |
| Application of two risk assessment methods on occupational hazard evaluation of comprehensive experimental building construction project of a research institute (87) | 2020 | Journal Article | Jing Wang | Beijing | Applied study | GBZ/T 298-2017, Singaporean model | Laboratory | Isopropyl alcohol, ethanol, acetone, hydrogen peroxide, xylene, formaldehyde, hydrochloric acid, sulfuric acid, mercury oxide, chloroform, acrylamide | Chinese |
| Application and comparison of two risk assessment methods in workplaces with dust exposure (88) | 2020 | Journal Article | Peixian Chen | Guangdong | Comparative study | ICMM model, GBZ/T 298-2017 | Automobile manufacturing, furniture manufacturing industry, coal wharf | Dust | Chinese |
| Quantitative differences between common occupational health risk assessment models (89) | 2020 | Journal Article | Qiuliang Xu | Zhejiang | Comparative study | ICMM model, Singaporean model, USEPA Model, Romanian model, Australian model, COSHH model | Leather production industry, wooden furniture manufacturing industry, printing and dyeing industry, printing industry, garment manufacturing industry | Chemical agents, dust | English |
| Quantitative comparison of six common occupational health risk assessment models for small printing companies (90) | 2020 | Journal Article | Qiuliang Xu | Zhejiang | Comparative study | ICMM model, Singaporean model, USEPA Model, Romanian model, Australian model, COSHH model | Printing industry | Benzene, toluene, xylene, ethyl acetate, acetone, butanone | Chinese |
| Application of four models of occupational health risk assessment of chemicals in a coating production enterprise (91) | 2020 | Journal Article | Qiuying Dong | Hebei | Applied study | ICMM model, Singaporean model, USEPA Model, OHR Index model | Paint production industry | Chemical agents such as toluene, xylene, ethyl acetate, methanol, acetone | Chinese |
| Wastewater treatment plant workers' exposure and methods for risk evaluation of their exposure (92) | 2020 | Journal Article | Rui Lu | Shaanxi | Applied study | USEPA Model | Wastewater treatment plant | Airborne bacteria and endotoxin | English |
| Occupational exposure to respirable dust from the coal-fired power generation process: sources, concentration, and health risk assessment (93) | 2020 | Journal Article | Ruipeng Tong | Beijing | Applied study | USEPA Model, Monte Carlo simulation | Thermal power generation industry | Dust | English |
| Application of three occupational health risk assessment methods in a lead-acid battery enterprise (94) | 2020 | Journal Article | Shishu Tang | Hubei | Applied study | GBZ/T 298-2017 | Lead-acid battery industry | Lead fume, lead dust, sulfuric acid | Chinese |
| Comparison between two methods for assessment of occupational health risk caused by coal dust (95) | 2020 | Journal Article | Siyu Zhang | Beijing | Comparative study | ICMM model, OHR Index model | Coal mining industry | Coal dust | Chinese |
| Risk assessment of workplace silica dust hazard in ceramic manufacturing enterprises: A comparison study of four risk assessment models (96) | 2020 | Journal Article | Tianzheng Li | Guangdong | Comparative study | GBZ/T 298-2017 | Ceramic manufacturing industry | Silica dust | Chinese |
| Application and comparison of four occupational health risk assessment methods in inorganic pigment manufacturing industry (97) | 2020 | Journal Article | Tingting Huo | Shanghai | Comparative study | ICMM model, Singaporean model | Inorganic pigment manufacturing industry | Dust; physical agents such as noise, high temperature; chemical agents such as sulfuric acid, sodium hydroxide | Chinese |
| Application of three semi-quantitative occupational health risk assessment methods in automobile manufacturing enterprises (98) | 2020 | Journal Article | Weiping Ye | Hubei | Applied study | GBZ/T 298-2017 | Automobile manufacturing industry | Welding fumes, grinding wheel grinding dust; chemical agents such as manganese and its inorganic compounds, nitrogen dioxide, toluene, xylene, methyl ethyl ketone, butyl acetate, butanol, isopropanol. | Chinese |
| Application of common occupational health risk assessment methods in vinyl chloride manufacturing factories (99) | 2020 | Journal Article | Yiwen Dong | Beijing | Applied study | GBZ/T 298-2017 | Chemical industry | Vinyl chloride | Chinese |
| Study on application of three occupational health risk assessment models in a coal-fired thermal power plant (100) | 2020 | Journal Article | Yue Gao | Jiangsu | Comparative study | ICMM model, Singaporean model, Romanian model | Thermal power generation industry | Noise, dust | Chinese |
| Application of risk assessment of dust occupational hazards during subway construction (101) | 2020 | Journal Article | Yun Lai | Jiangxi | Applied study | ICMM model | Construction industry | Dust | Chinese |
| Application of three occupational health risk assessment methods to noise risk assessment in a large equipment manufacturing enterprise (102) | 2020 | Journal Article | Zhiming Liang | Guangdong | Applied study | ICMM model, OHR Index model | Equipment manufacturing industry | Noise | Chinese |
| Comparison of four methods for assessing the risk of chemical hazards in the electrical appliance manufacturing industry (103) | 2020 | Journal Article | Zhiming Liang | Guangdong | Comparative study | ICMM model, GBZ/T 298-2017 | Appliance manufacturing industry | Toluene, n-hexane, cyclohexane, cyclohexanone, ethyl acetate, butyl acetate and acetone | Chinese |
| Application of fuzzy mathematical model in health risk assessment of occupational hazards in high-temperature refractory shed board manufacturing enterprises (104) | 2020 | Journal Article | Zhongan Xu | Guangdong | Applied study | Fuzzy model | Refractory material manufacturing industry | High temperature, noise, dust | Chinese |
| Application of various exposure assessment methods in the occupational health risk assessment of trichloroethylene in electroplating enterprises (105) | 2020 | Journal Article | Zhuandi Zhao | Guangdong | Applied study | GBZ/T 298-2017 | Electroplating industry | Trichloroethylene | Chinese |
| Occupational health risk assessment in a resin anchorage production workshop (106) | 2021 | Journal Article | Bin Feng | Shandong | Applied study | GBZ/T 298-2017 | Mining industry | Chemical agents such as styrene, dibutyl phthalate, phthalic anhydride | Chinese |
| Research and application of quantitative and semi-quantitative models for occupational disease hazard risk assessment in coatings industry (107) | 2021 | Journal Article | Biyin Chen | Guangdong | Applied study | GBZ/T 298-2017 | Paint production industry | Chemical agents such as benzene, toluene, xylene, n-butanol, butyl acetate, methyl methacrylate | Chinese |
| Application of three occupational health risk assessment methods in a large-scale integrated circuit manufacturing enterprise in Shenzhen City (108) | 2021 | Journal Article | Haili Qiu | Guangdong | Applied study | GBZ/T 298-2017 | Integrated circuit industry | Chemical agents | Chinese |
| Occupational health risk assessment of silicosis caused by silica dust exposure in non-ferrous metal mines (109) | 2021 | Thesis | Kai Liu | Beijing | Optimization study | ICMM model, OHR Index model | Non-ferrous metal mining industry | Dust | Chinese |
| Application of two occupational health risk assessment methods for dust hazard assessment in coal mines (110) | 2021 | Journal Article | Leping Qiu | Sichuan | Applied study | ICMM model, OHR Index model | Coal mining industry | Dust such as coal dust, silica dust | Chinese |
| A comparative study on application of three methods of occupational health risk assessment for alumina dust exposure workstations (111) | 2021 | Journal Article | Minghua Gu | Shanghai | Applied study | Singaporean model, Australian model, COSHH model | Abrasives manufacturing industry | Alumina dust | Chinese |
| Comparison of application of four occupational health risk assessment methods in electronic component and component manufacturing enterprises (112) | 2021 | Journal Article | Peng Zhang | Zhejiang | Comparative study | GBZ/T 298-2017 | Electronics manufacturing industry | Dust, benzene, toluene, xylene, styrene and tin dioxide | Chinese |
| Comparison of five occupational health risk assessment models applied to silica dust hazard in small open pits (113) | 2021 | Journal Article | Qiuliang Xu | Zhejiang | Comparative study | ICMM model, Romanian model, Australian model, COSHH model | Mining industry | Silica dust | Chinese |
| Comparison of application of different risk assessment methods in dust hazard assessment in gypsum board production enterprises (114) | 2021 | Journal Article | Runan Qin | Tianjin | Comparative study | GBZ/T 298-2017, OHR Index model | Construction material production industry | Gypsum dust, coal dust | Chinese |
| Application of improved comprehensive index method in risk assessment of FRP yacht manufacturing enterprises (115) | 2021 | Journal Article | Shihuai Zhang | Shandong | Optimization study | GBZ/T 298-2017 | Manufacturing industry | Styrene, dust | Chinese |
| Comparison of three methods applied in occupational health risk assessment of lead related posts (116) | 2021 | Journal Article | Shishu Tang | Hubei | Applied study | GBZ/T 298-2017 | Lead-acid battery industry | Lead | Chinese |
| Occupational health risk assessment of benzene, toluene, and xylene in Shanghai (117) | 2021 | Journal Article | Tongshuai Wang | Shanghai | Applied study | GBZ/T 298-2017 | Furniture manufacturing industry, metal products industry, printing industry | Benzene, toluene, and xylene | English |
| Application comparison of three kinds of occupational risk assessment methods in lighting industry (118) | 2021 | Journal Article | Wanxia Chen | Guangdong | Applied study | GBZ/T 298-2017 | Lighting fixture manufacturing industry | Chemical agents such as sulfur dioxide, mercury, butyl acetate, acetone | Chinese |
| Occupational dust hazards and risk assessment of coal-fired thermal power plants of different capacities - China, 2017-2019 (119) | 2021 | Journal Article | Xin Wang | Beijing | Applied study | OHR Index model | Coal-fired thermal power plants | Dust | English |
| Application of five methods in the occupational health risk assessment of workers exposed to welding fumes (120) | 2021 | Journal Article | Xue Zhao | Tianjin | Applied study | ICMM model, GBZ/T 298-2017 | Electric welding | Welding fumes | Chinese |
| Application of multi-risk assessment methods in occupational health risk assessment of silica dust hazards in workplace of stone processing industry (121) | 2021 | Journal Article | Xueyu Wang | Guangdong | Applied study | ICMM model, GBZ/T 298-2017 | Stone processing industry | Silica dust | Chinese |
| Application of two occupational health risk assessment methods in enterprises with 1-bromopropane production and utilization (122) | 2021 | Journal Article | Yi Zhang | Beijing | Applied study | Singaporean model, USEPA Model | Chemical industry | 1-Bromopropane | Chinese |
| Application of ICMM assessment model in occupational health risk assessment of a metal surface treatment enterprise (123) | 2021 | Journal Article | Yibing Qiu | Guangdong | Applied study | ICMM model | Metal surface treatment industry | Dust; chemical agents such as toluene, xylene, hydrochloric acid, sulfuric acid, phosphoric acid, sodium hydroxide, nitrogen dioxide | Chinese |
| Occupational health risk assessment of dust in cement production enterprises (124) | 2021 | Journal Article | Yong Niu | Beijing | Applied study | ICMM model, GBZ/T 298-2017 | Cement production industry | Cement dust | Chinese |
| Analysis of occupational health risk assessment results of two ferrous metal foundry enterprises (125) | 2021 | Journal Article | Yongquan Gu | Zhejiang | Applied study | ICMM model, USEPA Model | Ferrous metal foundry industry | Silica dust, grinding wheel dust; physical agents such as noise; chemical agents such as formaldehyde, benzene and xylene | Chinese |
| Application of different risk assessment methods in occupational health risk assessment in sewage treatment industry (126) | 2021 | Journal Article | Yuchao Feng | Guangdong | Applied study | ICMM model, GBZ/T 298-2017, USEPA Model, Australian model | Wastewater treatment industry | Dust; chemical agents such as ammonia, hydrogen sulfide | Chinese |
| Study on the application of the Technical Guidelines of occupational health risk assessment of hazardous chemical agents in workplace in lead-acid battery industry (127) | 2021 | Journal Article | Zhengmin Yu | Jiangsu | Applied study | GBZ/T 298-2017 | Lead-acid battery industry | Lead fume, lead dust; chemical agents such as sulfuric acid, benzene, toluene, xylene | Chinese |
| Occupational chemical hazard risk assessment of benzene and its analogies in storage tank areas of petrochemical enterprises based on risk matrix method (128) | 2021 | Journal Article | Zhenhang Xiao | Hubei | Optimization study | Fuzzy model | Petrochemical industry | Benzene | Chinese |
| Analysis the occupational health status of coal dust in 107 non-metallic mineral products enterprises in Sichuan Province and the applicability of semi-quantitative risk assessment model (129) | 2021 | Thesis | Zhichao Nie | Sichuan | Applied study | GBZ/T 298-2017 | Non-metallic mineral products industry | Coal dust | Chinese |
| Comparative study on common occupational health risk assessment methods in a paint manufacture (130) | 2021 | Journal Article | Zhiyang Zhou | Guangdong | Comparative study | GBZ/T 298-2017,Romanian model, Australian model | Paint production industry | Methyl methacrylate, toluene, n-hexane, benzene, cyclohexane, xylene, ethyl acetate, butyl methacrylate, n-butanol | Chinese |
| Volatile organic compound (VOC) emissions and health risk assessment in paint and coatings industry in the Yangtze River Delta, China (131) | 2021 | Journal Article | Ziwei Mo | Guangdong | Applied study | USEPA Model, Monte Carlo simulation | Paint and coatings industry | Volatile organic compounds | English |
| Application of three risk assessment methods to noise risk assessment in an automobile foundry enterprise (132) | 2022 | Journal Article | Beibei Xie | Hubei | Applied study | ICMM model, OHR Index model | Automobile manufacturing industry | Noise | Chinese |
| Application of three semi-quantitative risk assessment methods in brick and tile enterprises (133) | 2022 | Journal Article | Chan Yang | Sichuan | Applied study | GBZ/T 298-2017 | Brick and tile manufacturing industry | Silica dust, carbon monoxide | Chinese |
| Comparative study of risk assessment model and actual monitoring in the exposure evaluation of automobile manufacturers (134) | 2022 | Journal Article | Cheng Zhang | Hubei | Comparative study | GBZ/T 298-2017 | Automobile manufacturing industry | Dichloromethane | Chinese |
| Application of multiple methods in the assessment of occupational health risk of n-hexane in no-dust workplaces (135) | 2022 | Journal Article | Huanfeng Bian | Guangdong | Applied study | GBZ/T 298-2017 | Electronics manufacturing industry | n-hexane | Chinese |
| A comparative study of semi-quantitative and quantitative models in risk assessment of optical fiber manufacturing enterprises (136) | 2022 | Journal Article | Jingrong Liu | Hubei | Comparative study | GBZ/T 298-2017 | Optical fiber manufacturing industry | Chemical agents such as acetaldehyde, hydrogen chloride, chlorine, carbon monoxide, carbon dioxide | Chinese |
| Comparison of different semi-quantitative risk assessment methods applied in ammonia and hydrazine posts of power plants (137) | 2022 | Journal Article | Jintong He | Guangdong | Applied study | GBZ/T 298-2017, Singaporean model | Thermal power generation industry | Ammonia, hydrazine | Chinese |
| Application of three occupational health risk assessment methods in a coal-fired power generation enterprise in western region of Hainan Province (138) | 2022 | Journal Article | Lei Jin | Hainan | Applied study | Singaporean model, OHR Index model, Romanian model | Thermal power generation industry | Dust, physical agents | Chinese |
| Occupational health risk assessment of the benzene exposure industries: a comprehensive scoring method through 4 health risk assessment models (139) | 2022 | Journal Article | Ludi Zhang | Jiangsu | Optimization study | ICMM model, Singaporean model, USEPA Model | Benzene exposure industries | Benzene | English |
| Occupational health risk assessment of silica dust in small and micro non-coal mine enterprises in eight provinces in China from 2018 to 2020 (140) | 2022 | Journal Article | Penghui Fan | Beijing | Applied study | ICMM model, OHR Index model | Non-coal mining industry | Silica dust | Chinese |
| Risk assessment method and application of occupational hazards in operation exposed to aromatic mixture based PBPK model (141) | 2022 | Journal Article | Qinghua Guo | Tianjin | Applied study | PBPK model | Paint production industry | Benzene, toluene, xylene, ethylbenzene | Chinese |
| Applicability analysis of four occupational health risk assessment methods for jobs exposed to silica dust (142) | 2022 | Journal Article | Qiuying Dong | Hebei | Comparative study | ICMM model, Singaporean model, OHR Index model | Metal mining industry | Silica dust | Chinese |
| Application of multiple occupational health risk assessment models in occupation health risk prediction of trichloroethylene in the electroplating and electronics industries (143) | 2022 | Journal Article | Shibiao Su | Guangdong | Applied study | GBZ/T 298-2017, Singaporean model, USEPA Model, COSHH model | Electroplating and electronics industries | Trichloroethylene (TCE) | English |
| Application research on occupational health risk assessment technology of dust in brick and tile manufacturing industry (144) | 2022 | Journal Article | Ting Shi | Sichuan | Applied study | ICMM model, GBZ/T 298-2017, OHR Index model | Brick and tile manufacturing industry | Dust | Chinese |
| Study on applicability of five occupational health risk assessment methods in paint position of bus maintenance company (145) | 2022 | Journal Article | Weiping Ye | Hubei | Comparative study | GBZ/T 298-2017 | Public transportation repair and maintenance enterprise | Benzene, toluene, xylene and ethyl acetate | Chinese |

**List of included articles**

1. Lin S, Wang Z, Tang W, Wang M, Lan Y, Wang P. A Preliminary Study on the Evaluation Method of Occupational Hazard Risk Index. *Chinese Journal of Industrial Hygiene and Occupational Diseases* (2006) (12):769-71. doi: (In Chinese).

2. Huang D, Guan S, Bo Y, Chen H. Health Risk Assessment of Occupational Exposure to Toxic Substances. *Chinese Journal of Industrial Hygiene and Occupational Diseases* (2007) 25(08):512-3. doi: (In Chinese).

3. Huang DY, Zhang J, Liu M, Ieee, editors. Application of a Health Risk Classification Method to Assessing Occupational Hazard in China. *2009 3rd International Conference on Bioinformatics and Biomedical Engineering, vols 1-11*; 2009; 3rd International Conference on Bioinformatics and Biomedical Engineering.

4. Huang D, Liu M, Zhang J, Wang Y, editors. Research on Risk Assessment Based on Monte Carlo Simulation and Dose-Response Multistage Model. *2010 3rd International Conference on Biomedical Engineering and Informatics (BMEI 2010), vols 1-7*; 2010; 2010 3rd International Conference on Biomedical Engineering and Informatics (BMEI 2010).

5. Ji Q, Li N. Application of Lec Evaluation Method in Risk Assessment of Occupational Disease Hazards in Construction Projects. *Occupational Health and Emergency Rescue* (2011) 29(05):260-2. doi: 10.16369/j.oher.issn.1007-1326.2011.05.009 (In Chinese).

6. Zhu-Wu Z, Yong-Kui S, Guang-Peng Q, Ping-Yong B. Research on the Occupational Hazards Risk Assessment in Coal Mine Based on the Hazard Theory. *Procedia Engineering* (2011) 26:2157-64. doi: 10.1016/j.proeng.2011.11.2420.

7. Xue M, Yang Y, Ruan J, Xu Z. Assessment of Noise and Heavy Metals (Cr, Cu, Cd, Pb) in the Ambience of the Production Line for Recycling Waste Printed Circuit Boards. *Environmental Science & Technology* (2012) 46(1):494-9. doi: 10.1021/es202513b.

8. Jiang C, Yang Z, Zhang X. Application of Risk Assessment Method for Occupational Hazard in Dye Chemical Enterprise. *Chinese Journal of Health Laboratory Technology* (2013) 23(14):2961-3+7. doi: (In Chinese).

9. Wang S, Zhang M, Guoqin J, Lian L, Li M. An Application Study of Australian Occupational Risk Assessment Model in a Certain Battery Production Enterprise. *Preventive Medicine* (2013) 25(12):8-11+48. doi: 10.19485/j.cnki.issn1007-0931.2013.12.003 (In Chinese).

10. Wang Z, Zheng W, Xiao G. Occupational Disease Hazards Evaluation in an Enterprise by a Semi-Quantitative Risk Assessment Method.*Journal of Environmental and Occupational Medicine* (2013) 30(09):686-9. doi: 10.13213/j.cnki.jeom.2013.09.005 (In Chinese).

11. Leng B, Bian G, Wang A, Wang Q, Zhang M. Application of U.S. Epa Inhalation Risk Model to Occupational Health Risk Assessment of Wooden Furniture Manufacturing Factories. *Journal of Environmental and Occupational Medicine* (2014) 31(11):858-62. doi: 10.13213/j.cnki. jeom2014.0208 (In Chinese).

12. Wen C, Chen J, Li R, Li L, Wu X. Application of Three Risk Assessment Methods on Chemicals Risk Assessment and Management. *Chinese Journal of Public Health Engineering* (2014) 13(03):184-6+9. doi: (In Chinese).

13. Zhou L, Zhang M, Yuan W, Zou H. A Study on Application of Inhalation Risk Assessment Model of Usepa in Occupational Health Risk Assessment. *Preventive Medicine* (2014) 26(02):109-13+27. doi: 10.19485/j.cnki.issn1007-0931.2014.02.001 (In Chinese).

14. Zhou P, Guo J, Zhou X, Zhang W, Liu L, Liu Y, et al. Pm2.5, Pm10 and Health Risk Assessment of Heavy Metals in a Typical Printed Circuit Boards Manufacturing Workshop. *Journal of Environmental Sciences* (2014) 26(10):2018-26. doi: 10.1016/j.jes.2014.08.003.

15. Ye S, Peng X, Hu D, Zhao X, Yu Ra. Health Risk of Shenzhen Gas Station Workers Exposed to Mtbe: A Primary Study Based on Pbpk Model. *Journal of Environment and Health* (2014) 31(12):1076-9. doi: 10.16241/j.cnki.1001-5914.2014.12.008 (In Chinese).

16. Chen S, Tan Q, Liang H, Dong X, Guo Y, He M, et al. Application of Lec Method in Occupational Health Risk Assessment of Ceramic Enterprise. *Occupation and Health* (2014) 30(09):1157-9. doi: 10.13329/j.cnki.zyyjk.2014.09.035 (In Chinese).

17. Li X. A Study on Application of Five Risk Assessment Methods for Occupational Health [master's thesis]. Zhejiang: Zhejiang University (2014). doi: (In Chinese).

18. Yuan W, Leng P, Zhou L, Zou H, Zhang M. Comparative Study on Occupational Risk Assessment Using Two Foreign Models.*Journal of Environmental and Occupational Medicine* (2015) 32(01):51-5. doi: 10.13213/j.cnki.jeom2015.14292 (In Chinese).

19. Yan Y, Peng L, Cheng N, Bai H, Mu L. Health Risk Assessment of Toxic Vocs Species for the Coal Fire Well Drillers. *Environmental Science and Pollution Research* (2015) 22(19):15132-44. doi: 10.1007/s11356-015-4729-7.

20. Hu D, Yang J, Liu Y, Zhang W, Peng X, Wei Q, et al. Health Risk Assessment for Inhalation Exposure to Methyl Tertiary Butyl Ether at Petrol Stations in Southern China. *International Journal of Environmental Research and Public Health* (2016) 13(2). doi: 10.3390/ijerph13020204.

21. Gao H. A Study on Application and Modification of Three Occupational Health Risk Assessment Models [master's thesis]. Xinjiang: Shihezi University (2016). doi: (In Chinese).

22. Fu H, Yu A, Zhang L, Wang Q, Ruan X, Yang Y, et al. Application of Semi-Quantitative Risk Assessment Model in Glyphosate Industry in Singapore. *Chinese Preventive Medicine* (2016) 17(12):916-20. doi: 10.16506/j.1009-6639.2016.12.009 (In Chinese).

23. Xie H, Zhang M, Zhou L, Quan Z, Chen R, Zhu J. Application of Two Risk Assessment Models to Printing Industry.*Journal of Environmental and Occupational Medicine* (2016) 33(01):29-33. doi: 10.13213/j.cnki.jeom2016.15212 (In Chinese).

24. Leng P, Wang Q, Wang A, Bian G, Li X, Zhang M. Icmm Risk Assessment Technology-Based Research on Quantitive and Qualitative Assessment of a Electroplating Enterprise on Occupational Health Risks. *Chinese Journal of Public Health Engineering* (2016) 15(06):544-9. doi: (In Chinese).

25. Min Q. Application of Icmm Occupational Health Risk Assessment Model in Evaluation of Occupational Risk of a Refractory Enterprise.*Chinese Journal of Industrial Medicine* (2016) 29(06):452-4. doi: 10.13631/j.cnki.zggyyx.2016.06.023 (In Chinese).

26. Yu X, Han L, Xie K, He L, Zhang M. Romanian Method for Risk Assessment of Occupational Accidents and Diseases Application Effect in a Precious Metal Smelter. *Preventive Medicine* (2016) 28(02):186-8+91. doi: 10.19485/j.cnki.issn1007-0931.2016.02.027 (In Chinese).

27. Tang X, Zou Y, Lu L. Application of Lec Method in Occupation Health Risk Assessment of Toner Production Enterprise. *Chinese Journal of Public Health Management* (2016) 32(05):652-4. doi: 10.19568/j.cnki.23-1318.2016.05.024 (In Chinese).

28. Cao Y, Qi Z. Application of Graham Method in Health Risk Assessment of Occupational Hazards. *Occupational Health and Emergency Rescue* (2016) 34(06):453-5+66. doi: 10.16369/j.oher.issn.1007-1326.2016.06.005 (In Chinese).

29. Wu Z, Xu B, Jiang H, Zheng M, Zhang M, Zhao W, et al. Application of Three Risk Assessment Models in Occupationali Health Riisk Assessment of Dimethylformamide. *Chinese Journal of Industrial Hygiene and Occupational Diseases* (2016) 34(08):576-80. doi: 10.3760/cma.j.issn.1001-9391.2016.08.004 (In Chinese).

30. Wang A, Leng P, Li X, Wang Q, Gu S, Zhang M. Application of Two Risk Assessment Methods to Occupational Health Risk Assessment in a Ferrous Metal Foundry.*Journal of Environmental and Occupational Medicine* (2017) 34(10):909-13. doi: 10.13213/j.cnki.jeom.2017.17209 (In Chinese).

31. Wu B, Zhang Y, Chen Y. Study on Application of Two Risk Assessment Methods in Coal Dust Occupational Health Risk Assessment. *Chinese Journal of Industrial Hygiene and Occupational Diseases* (2017) 35(04):276-9. doi: 10.3760/cma.j.issn.1001-9391.2017.04.009 (In Chinese).

32. Huang G, Zhao M, Wu F, Tang S. Application of Semi-Quantitative Risk Assessment Technology in Electronic Enterprises. *Safety & Security* (2017) 38(04):6-8. doi: (In Chinese).

33. Bian G, Wang A, Li X, Zhang M, Zhang Z. A Comparative Study on the Application of Different Methods of Occupation Health Risk Assessment in Small Furniture Manufacturing Industry. *Preventive Medicine* (2017) 29(10):1003-8. doi: 10.19485/j.cnki.issn1007-0931.2017.10.008 (In Chinese).

34. Zhang J, Wu Z, Li F, Liu C, Yu C. Health Risk Assessment Method for Chinese Stone Quarrying Based on Icmm Evaluation Method. *Environmental Engineering* (2017) 35(05):141-4+8. doi: 10.13205/j.hjgc.201705030 (In Chinese).

35. Han L, Yu X, Xie K, Wang S, Tao J, Xu Z, et al. Application of Epa Inhalation Risk Assessment Model in Mercury Occupational Health Risk in Fluorescent Lamp Manufacturing Enterprises. *Preventive Medicine* (2017) 29(06):625-8. doi: 10.19485/j.cnki.issn1007-0931.2017.06.023 (In Chinese).

36. Zhou L, Zhang M, Zou H, Yuan W, Quan Z. Application of Two Health Risk Assessment Models in the Occupational Health Risk Assessment of Chemicals in Different Industries. *Preventive Medicine* (2017) 29(12):1217-22. doi: 10.19485/j.cnki.issn1007-0931.2017.12.007 (In Chinese).

37. Li M, Wang S, Jiang G, Zhang M. A Study on the Application of Ｒomania Risk Assessment Method of Occupational Accidents and Diseases in a Certain Fluorescent Lamp Manufacture Enterprise. *Preventive Medicine* (2017) 29(02):146-9+54. doi: 10.19485/j.cnki.issn1007-0931.2017.02.011 (In Chinese).

38. Wang Q, Wang Y, Liu Z, Qiu Y, Ji Q, Wang T, et al. Comparing the Risk of Formaldehyde in a Plywood Manufacturing Factory with Two Risk Assessment Methods. *China Occupational Medicine* (2017) 44(03):309-12. doi: 10.11763/j.issn.2095-2619.2017.03.012 (In Chinese).

39. Liu T, Zhang P, Ma L, Zhang C, Zhu J, Zhang M. Occupational Semi-Quantitative Risk Assessment in a Crane Manufacturing Enterprise. *Preventive Medicine* (2017) 29(04):347-50+54. doi: 10.19485/j.cnki.issn1007-0931.2017.04.006 (In Chinese).

40. Luan Y, Zhang M, Zou H, Quan Z. A Study on Application of Semi-Quantitative Risk Assessment Models in Furniture Industry *Preventive Medicine* (2017) 29(08):770-6. doi: 10.19485/j.cnki.issn1007-0931.2017.08.004 (In Chinese).

41. Wu B, Li M, Cheng X, Hou F, Xing C. Occupational Health Risk Assessment of Benzene Exposure among Enterprises.*Chinese Journal of Public Health* (2018) 34(05):755-8. doi: 10.11847/zgggws1118203 (In Chinese).

42. Wu B, Cheng X, Zhang G, Li M, Hou F, Xing C. Assessment on Occupational Health Risks of 1,3-Butadiene Exposure by Two Risk Assessment Models. *Occupation and Health* (2018) 34(07):865-8. doi: 10.13329/j.cnki.zyyjk.2018.0238 (In Chinese).

43. Tian F, Zhang M, Zhou L, Lou H, Wang A, Hao M. Qualitative and Quantitative Differences between Common Occupational Health Risk Assessment Models in Typical Industries. *Journal of Occupational Health* (2018) 60(5):337-47. doi: 10.1539/joh.2018-0039-OA.

44. Ye F, Yang L, Zhang W, Su X, Liu B. Risk Assessment of Pneumoconiosis in the Ceramic Production and Refractory Manufacturing. *Occupation and Health* (2018) 34(14):1873-6+80. doi: 10.13329/j.cnki.zyyjk.2018.0521 (In Chinese).

45. Han F, Chen Y, Wu B, Kang N, Zhang S. Occupational Health Risk Assessment of Coal Industry Chain. *Chinese Journal of Industrial Hygiene and Occupational Diseases* (2018) 36(04):291-4. doi: 10.3760/cma.j.issn.1001-9391.2018.04.015 (In Chinese).

46. Bian H, Hu W, Zhang H, Chen L, Chen Z, Tian Y, et al. Comparative Study on Application of Different Occupational Health Risk Assessment Methods in Lead-Acid Battery Production Industry. *China Occupational Medicine* (2018) 45(06):713-8. doi: 10.11763/j.issn.2095-2619.2018.06.0101 (In Chinese).

47. Zhang J, Cai Y, Li F, Wu Z. Comparison of Occupational Health Risk Assessment of an Electronic Enterprise Based on Epa Method and Occupational Disease Work Classification Method. *Journal of Safety and Environment* (2018) 18(05):1692-8. doi: 10.13637/j.issn.1009-6094.2018.05.008 (In Chinese).

48. Zhang J, Wu Z, Li F, Yu C, Peng X. Comparison of Application Effects of Several Occupational Health Risk Assessment Methods in Quarrying Industry. *Journal of Safety and Environment* (2018) 18(01):23-7. doi: 10.13637/j.issn.1009-6094.2018.01.005 (In Chinese).

49. Chen L, Qian X, Liu J, Hu W, Yang H, Zhang H. Application of Icmm Occupational Health Risk Assessment Modelin Evaluation of Occupational Risk of a Lead-Acid Battery Enterprise. *Chinese Journal of Industrial Hygiene and Occupational Diseases* (2018) 36(04):298-301. doi: 10.3760/cma.j.issn.1001-9391.2018.04.017 (In Chinese).

50. Chen L, Qian X, Zhao D, Feng X, Da W, Hu W, et al. Occupational Hazards in a Lead—Acid Battery Enterprise：a Comparison Study of Three Health Risk Assessment Methods.*Chinese Journal of Public Health* (2018) 34(06):849-53. doi: 10.11847/zgggws1119044 (In Chinese).

51. Chen L, Qian X, Zhao Q, Hu W, Yu W, Wang X, et al. Application of Singapore Occupational Hazards Semi—Quantitative Risk Assessment Method in a Lead—Acid Battery Enterprise. *China Occupational Medicine* (2018) 45(01):123-5. doi: 10.11763/j.issn.2095-2619.2018.01.027 (In Chinese).

52. Zhang M, Zhou Z, Ren F, Jiang H, Xu B, Xu C, et al. Application of Three Risk Assessment Models in Occupational Health Risk Assessment of Toluene. *Occupation and Health* (2018) 34(23):3180-5. doi: 10.13329/j.cnki.zyyjk.2018.0890 (In Chinese).

53. Su M, Sun R, Zhang X, Wang S, Zhang P, Yuan Z, et al. Assessment of the Inhalation Risks Associated with Working in Printing Rooms: A Study on the Staff of Eight Printing Rooms in Beijing, China. *Environmental Science and Pollution Research* (2018) 25(17):17137-43. doi: 10.1007/s11356-018-1802-z.

54. Chen P, Zhang H, Ling W, Tang S, Xu S, Yang Y, et al. Comparison of Two Methods of Evaluating Occupational Health Risk in a Shoemaking Enterprise. *China Occupational Medicine* (2018) 45(01):55-9. doi: 10.11763/j.issn.2095-2619.2018.01.011 (In Chinese).

55. Zhang P, Liu T, Li H, Zhang C, Ma L, Zhang M. Applied Study and Case Report of Comparison and Application of Two Occupational Health Risk Assessment Methods in Chair Furniture Manufacturing Enterprises. *Preventive Medicine* (2018) 30(02):158-62. doi: 10.19485/j.cnki.issn2096-5087.2018.02.012 (In Chinese).

56. Leng P, Li X, Wang Q, Mao G, Wang A. Occupational Health Risk Assessment for Workers Exposed to Low Concentration of Benzene.*Journal of Environmental and Occupational Medicine* (2018) 35(11):985-9. doi: 10.13213/j.cnki.jeom.2018.18252 (In Chinese).

57. Ding S, Hu Z, Wu C. Application of Two Risk Assessment Methods on Occupational Health Risk Assessment of Wet Sand Casting Molding Process. *Occupation and Health* (2018) 34(24):3324-8. doi: 10.13329/j.cnki.zyyjk.2018.0933 (In Chinese).

58. Cao S. Application of Two Occupational Health Risk Assessment Models in an Automobile-Component Manufactory in Fengxian District of Shanghai. *Occupation and Health* (2018) 34(20):2740-4. doi: 10.13329/j.cnki.zyyjk.2018.0730 (In Chinese).

59. Huang T, Su S, Jin Y, Lu J, Wu M. Application of Occupational Health Risk Assessment Model of International of Council on Mining and Metals in Calcium Carbonate Production Enterprises *China Occupational Medicine* (2018) 45(06):753-7. doi: 10.11763/j.issn.2095-2619.2018.06.019 (In Chinese).

60. Li X, Ding J, Liu M, Xu H, Su S, Hu S. Application Research of Three Risk Assessment Methods to Organic Solvents in Painting Produced Industry. *Preventive Medicine* (2018) 30(08):794-8. doi: 10.19485/j.cnki.issn2096-5087.2018.08.009 (In Chinese).

61. Tian Y, Liu K, Wu L, Zhu Z, Dai Z, Wang L, et al. Application of Three Models for Occupational Health Risk Assessment to a Transformer Factory in Shenzhen City. *Occupation and Health* (2018) 34(18):2449-52. doi: 10.13329/j.cnki.zyyjk.2018.0688 (In Chinese).

62. Tian Y, Liu K, Wu L, Lihua W, Dai Z, Feng J, et al. Comparison of the Application of Three Occupational Health Risk Assessment Models in Battery Manufacturers. *Preventive Medicine* (2018) 30(12):1248-51. doi: 10.19485/j.cnki.issn2096-5087.2018.12.015 (In Chinese).

63. Zou Y, Lu L, Tang Xo, Wen W, Lin H, Su S. Comparison of Qualitative and Semi-Quantitative Occupational Health Risk Assessment Methods in an Adhesive Manufacturer. *China Occupational Medicine* (2018) 45(06):770-4+8. doi: 10.11763/j.issn.2095-2619.2018.06.023 (In Chinese).

64. Cai Y, Li F, Zhang J, Wu Z. Occupational Health Risk Assessment in the Electronics Industry in China Based on the Occupational Classification Method and Epa Model. *International Journal of Environmental Research and Public Health* (2018) 15(10). doi: 10.3390/ijerph15102061.

65. Liang Z, Fu F, Li L, Jin Y, Lin H, Ceng Q, et al. Comparison of Multiple Risk Assessment Methods for Occutaptional Health Risk Assessment for Aluminum Dust Post. *China Occupational Medicine* (2018) 45(06):766-9. doi: 10.11763/j.issn.2095-2619.2018.06.022 (In Chinese).

66. Lin Z, Lin H, Xie D, Lin X, Huang S, Chen X. Occupational Health Risk Assessment on 1,2-Dichloroethane in Toy Manufacturers in Shantou City. *China Occupational Medicine* (2018) 45(01):111-4. doi: 10.11763/j.issn.2095-2619.2018.01.024 (In Chinese).

67. Wang A, Pengbo L, Xiaohai L, Guochuan M, Guozhang X. Occupational Health Risk Assessment of Low Concentrations Benzene Toluene and Xylenes. *Chinese Journal of Industrial Hygiene and Occupational Diseases* (2019) 37(8):627-32. doi: 10.3760/cma.j.issn.1001-9391.2019.08.018 (In Chinese).

68. Liu B. Icmm Risk Assessment Model Assessing a Coal-Fired Power Plants’ Occupational Health Risk. *Henan Journal of Preventive Medicine* (2019) 30(01):81-5. doi: 10.13515/j.cnki.hnjpm. 1006-8414.2019.01.029 (In Chinese).

69. Wang D. Improvement and Application Research on Evaluation Method of Coal Mine Dust Hazard Degree [master's thesis]. Beijing: Capital University of Economics and Business (2019). doi: 10.27338/d.cnki.gsjmu.2019.000742 (In Chinese).

70. Li H, Song S, Li T, Zhao S, Qian X, Tao X. Application of Two Risk Assessment Methods in Occupational Health Risk of 2-Butoxyethonal. *China Occupational Medicine* (2019) 46(01):46-9. doi: 10.11763/j.issn.2095-2619.2019.01.009 (In Chinese).

71. Bian H, Kang N, Dong Y, Qiu L, Hu W. Comparative Study of Three Semi-Quantitative Risk Assessment Methods in Risk Classification of Silica Dusts Exposed Posts.*Chinese Journal of Industrial Medicine* (2019) 32(03):167-71. doi: 10.13631/j.cnki.zggyyx.2019.03.002 (In Chinese).

72. Chen H, Yan X, Chen Y, Zhao L, Li L. A Comparative Study of Three Risk Assessment Methods on Evaluation of Risk Rating for Dust Hazard in an Offshore Oil Engineering Equipment Manufacturing Project of Guangdong Province. *Occupation and Health* (2019) 35(09):1171-5. doi: 10.13329/j.cnki.zyyjk.2019.0310 (In Chinese).

73. Ding J. Application Research of Four Occupational Health Risk Assessment Methods in Chemical Enterprises [master's thesis]. Guangdong: Guangdong Pharmaceutical University (2019). doi: 10.27690/d.cnki.ggdyk.2019.000170 (In Chinese).

74. Ding J, Su S, Jin Y, Liu M, Tan W. Comparison of Three Health Risk Assessment Methods for Organic Solvents in Furniture Manufacturing Enterprises. *Preventive Medicine* (2019) 31(04):400-4. doi: 10.19485/j.cnki.issn2096-5087.2019.04.019 (In Chinese).

75. Wang L, Zhang L, Sun Y, Cheng H, Zhang F, Shao H. Application of 3 Methods on Occupational Health Risk Assessment of Methyl Isoamyl Ketone. *China Occupational Medicine* (2019) 46(06):732-6. doi: 10.11763/j.issn.2095-2619. 2019.06.017 (In Chinese).

76. Chen L, Cen Z, Ma W, Jin Y, Shu Y, Liu Y, et al. Application of Three Methods in Occupational Health Risk Assessment of Automobile 4s Shop. *Chinese Journal of Industrial Hygiene and Occupational Diseases* (2019) (11):866-7-8-9-70. doi: 10.3760/cma.j.issn.1001-9391.2019.11.018 (In Chinese).

77. Tong R, Cheng M, Ma X, Yang Y, Liu Y, Li J. Quantitative Health Risk Assessment of Inhalation Exposure to Automobile Foundry Dust. *Environmental Geochemistry and Health* (2019) 41(5):2179-93. doi: 10.1007/s10653-019-00277-8.

78. Zou S, Dong Q, Zhao C, Li J. Application of Icmm Occupation Health Risk Assessment Model in Occupational Hazard Assessment of a Coking Plant in Chengde City. *Occupation and Health* (2019) 35(08):1009-13+18. doi: 10.13329/j.cnki.zyyjk.2019.0268 (In Chinese).

79. Huang T, Huang R. Application of Fuzzy Mathematics Method in Risk Assessment of Occupational Disease Hazards in Wooden Furniture Manufacturing Enterprises. *Occupation and Health* (2019) 35(02):145-8+52. doi: 10.13329/j.cnki.zyyjk.2019.0042 (In Chinese).

80. Yan W, Liu J, Chen K, Wang J, Su S, Li R. Application of Three Semi-Quantitative Risk Assessment Methods in Electroplating Enterprises. *Chinese Journal of Industrial Hygiene and Occupational Diseases* (2019) (06):420-5. doi: 10.3760/cma.j.issn.1001-9391.2019.06.004 (In Chinese).

81. Li X, Huang J, Jiang F, Wen J, Mei L, Chen F, et al. Application of Singapore Semi-Quantitative Risk Assessment Model for Occupational Exposure to Chemical Toxicants in Automotive Manufacturing Industry. *Occupation and Health* (2019) 35(18):2469-72+76. doi: 10.13329/j.cnki.zyyjk.2019.0661 (In Chinese).

82. Liu X, Zhang F, Zhu B. Application of Occupational Health Risk Assessment Model in Glyphosate Production Enterprises. *Chinese Journal of Industrial Hygiene and Occupational Diseases* (2019) (07):525-8. doi: 10.3760/cma.j.issn. 1001-9391.2019.07.010 (In Chinese).

83. Yang Y, Min X, Jian C, Shihao T, Shaoxiong X, Yuan Z, et al. Application of Semi-Quantitative Risk Assessment Method in Occupational Health Risk Assessment of Wooden Furniture Manufacturing Enterprises. *Chinese Journal of Industrial Hygiene and Occupational Diseases* (2019) 37(5):369-73. doi: 10.3760/cma.j.issn.1001-9391.2019.05.011 (In Chinese).

84. Meng C, Qiao P, Liang X, Meng Z, Liu Q, Shi J, et al. Application of Ccupational Health Risk Assessment Method in Dioctyl Phthalate Producing Enterprise *Chinese Journal of Industrial Hygiene and Occupational Diseases* (2020) 38(06):463-5. doi: 10.3760/cma.j.cn121094-20190325-00074 (In Chinese).

85. Wang H, Qiu L, Yin L. Application of Occupational Health Risk Assessment Method in Lead Zinc Mining Industry. *Occupational Health and Damage* (2020) 35(01):10-5+9. doi: (In Chinese).

86. Lu J, Tang J, Deng X, Xue L, Liu X. Occupational Health Risk Assessment of a Lead -Acid Battery Enterprise Based on Two Models of Icmm Occupational Health Risk Assessment. *Occupational Health and Emergency Rescue* (2020) 38(05):482-6. doi: 10.16369/j.oher.issn.1007-1326.2020.05.011 (In Chinese).

87. Wang J, Song H, Gao M. Application of Two Risk Assessment Methods on Occupational Hazard Evaluation of Comprehensive Experimental Building Construction Project of a Research Institute. *Occupation and Health* (2020) 36(21):2891-4+8. doi: 10.13329/j.cnki.zyyjk.2020.0793 (In Chinese).

88. Chen P, Zhang H, Yang Y, Liu Y. Application and Comparison of Two Risk Assessment Methods in Workplaces with Dust Exposure. *Occupational Health and Emergency Rescue* (2020) 38(04):352-6. doi: 10.16369/j.oher.issn.1007-1326.2020.04.007 (In Chinese).

89. Xu Q, Yu F, Li F, Zhou H, Zheng K, Zhang M. Quantitative Differences between Common Occupational Health Risk Assessment Models. *Journal of Occupational Health* (2020) 62(1). doi: 10.1002/1348-9585.12164.

90. Xu Q, Zhang M, Zou H, Yuan W, Li F. Quantitative Comparison of Six Common Occupational Health Risk Assessment Models for Small Printing Companies. *Journal of Environmental and Occupational Medicine* (2020) 37(02):131-7. doi: 10.13213/j.cnki.jeom.2020.19624 (In Chinese).

91. Dong Q, Li J, Hao S, Zhang J, Xu P, Zhao J, et al. Application of Four Models of Occupational Health Risk Assessment of Chemicals in a Coating Production Enterprise. *Chinese Journal of Industrial Medicine* (2020) 33(01):71-4. doi: 10.13631/j.cnki.zggyyx.2020.01.024 (In Chinese).

92. Lu R, Frederiksen MW, Uhrbrand K, Li Y, Ostergaard C, Madsen AM. Wastewater Treatment Plant Workers' Exposure and Methods for Risk Evaluation of Their Exposure. *Ecotoxicology and Environmental Safety* (2020) 205. doi: 10.1016/j.ecoenv.2020.111365.

93. Tong R, Liu J, Ma X, Yang Y, Shao G, Li J, et al. Occupational Exposure to Respirable Dust from the Coal-Fired Power Generation Process: Sources, Concentration, and Health Risk Assessment. *Archives of Environmental and Occupational Health* (2020) 75(5):260-73. doi: 10.1080/19338244.2019.1626330.

94. Tang S, Liang J, Chen Z, Dai X, Zhang C, Mao G. Application of Three Occupational Health Risk Assessment Methods in a Lead-Acid Battery Enterprise. *Journal of Hygiene Research* (2020) 49(02):298-301. doi: 10.19813/j.cnki.weishengyanjiu.2020.02.022 (In Chinese).

95. Zhang S, Han F, Ye M, Chen Y. Comparison between Two Methods for Assessment of Occupational Health Risk Caused by Coal Dust. *Occupational Health and Emergency Rescue* (2020) 38(03):238-41. doi: 10.16369/j.oher.issn.1007-1326.2020.03.006 (In Chinese).

96. Li T, Guan Y, Chen H, Lin Y, Feng J, Zhou W. Risk Assessment of Workplace Silica Dust Hazard in Ceramic Manufacturing Enterprises: A Comparison Study of Four Risk Assessment Models. *Journal of Environmental and Occupational Medicine* (2020) 37(02):138-43. doi: 10.13213/j.cnki.jeom.2020.19558 (In Chinese).

97. Huo T, Shang Y, Guo M. Application and Comparison of Four Occupational Health Risk Assessment Methods in Inorganic Pigment Manufacturing Industry. *Occupational Health and Emergency Rescue* (2020) 38(04):346-51. doi: 10.16369/j.oher.issn.1007-1326.2020.04.006 (In Chinese).

98. Ye W, Zhang C, Liang J, Mao G, Chen Z. Application of Three Semi-Quantitative Occupational Health Risk Assessment Methods in Automobile Manufacturing Enterprises. *Journal of Environmental and Occupational Medicine* (2020) 37(02):150-6. doi: 10.13213/j.cnki.jeom.2020.19596 (In Chinese).

99. Dong Y, Bian H, Wang X, Hu W. Application of Common Occupational Health Risk Assessment Methods in Vinyl Chloride Manufacturing Factories. *Journal of Environmental and Occupational Medicine* (2020) 37(08):797-803. doi: 10.13213/j.cnki.jeom.2020.19870 (In Chinese).

100. Gao Y, Yu Z, Liu X, Zhang H, Zhang C. Study on Application of Three Occupational Health Risk Assessment Models in a Coal-Fired Thermal Power Plant. *Shanghai Journal of Preventive Medicine* (2020) 32(11):888-94. doi: 10.19428/j.cnki.sjpm.2020.19975 (In Chinese).

101. Lai Y, Ai L, Tian Y, Zhang L, Liu Y. Application of Risk Assessment of Dust Occupational Hazards During Subway Construction. *Chinese Journal of Industrial Hygiene and Occupational Diseases* (2020) 38(04):297-9. doi: 10.3760/cma.j.cn121094-20190619-00256 (In Chinese).

102. Liang Z, Ceng Q, Deng Y, Li L, Yu J, Liang X, et al. Application of Three Occupational Health Risk Assessment Methods to Noise Risk Assessment in a Large Equipment Manufacturing Enterprise. *Journal of Environmental and Occupational Medicine* (2020) 37(02):144-9. doi: 10.13213/j.cnki.jeom.2020.19546 (In Chinese).

103. Liang Z, Ceng Q, Deng Y, Li L, Yu J, Zhong X, et al. Comparison of Four Methods for Assessing the Risk of Chemical Hazards in the Electrical Appliance Manufacturing Industry. *Preventive Medicine* (2020) 32(03):310-4. doi: 10.19485/j.cnki.issn2096-5087.2020.03.025 (In Chinese).

104. Xu Z, Wu L, Tang W. Application of Fuzzy Mathematical Model in Health Risk Assessment of Occupational Hazards in High-Temperature Refractory Shed Board Manufacturing Enterprises. *Occupational Health and Emergency Rescue* (2020) 38(03):246-9+61. doi: 10.16369/j.oher.issn.1007-1326.2020.03.008 (In Chinese).

105. Zhao Z, Zhang S, Chen J, Fu X, Zhu L, Su S. Application of Various Exposure Assessment Methods in the Occupational Health Risk Assessment of Trichloroethylene in Electroplating Enterprises. *Chinese Journal of Industrial Hygiene and Occupational Diseases* (2020) 38(10):782-6. doi: 10.3760/cma.j.cn121094-20191103-00514 (In Chinese).

106. Feng B, Zhang H, Zhang F, Zhang L, Li C, Wang H, et al. Occupational Health Risk Assessment in a Resin Anchorage Production Workshop. *China Occupational Medicine* (2021) 48(05):534-7. doi: 10.20001/j.issn.2095-2619.20211868 (In Chinese).

107. Chen B, Li W, Xu Q, Tan J. Research and Application of Quantitative and Semi-Quantitative Models for Occupational Disease Hazard Risk Assessment in Coatings Industry. *Chinese Journal of Public Health Engineering* (2021) 20(05):711-5. doi: 10.19937/j.issn.1671-4199.2021.05.003 (In Chinese).

108. Qiu H, Li T, Xiang Y, Wang X, Guan Y, Zhou W. Application of Three Occupational Health Risk Assessment Methods in a Large-Scale Integrated Circuit Manufacturing Enterprise in Shenzhen City. *Occupation and Health* (2021) 37(12):1603-8. doi: 10.13329/j.cnki.zyyjk.2021.0399 (In Chinese).

109. Liu K. Occupational Health Risk Assessment of Silicosis Caused by Silica Dust Exposure in Non-Ferrous Metal Mines [master's thesis]. Beijing: Chinese Center for Disease Control and Prevention (2021). doi: 10.27511/d.cnki.gzyyy.2021.000046 (In Chinese).

110. Qiu L, Ye M, Yin L, Zhang C. Application of Two Occupational Health Risk Assessment Methods for Dust Hazard Assessment in Coal Mines. *Occupation and Health* (2021) 37(24):3313-5+20. doi: 10.13329/j.cnki.zyyjk.2021.0796 (In Chinese).

111. Gu M, Xu X, Zhang M, Li Y. A Comparative Study on Application of Three Methods of Occupational Health Risk Assessment for Alumina Dust Exposure Workstations. *Journal of Environmental and Occupational Medicine* (2021) 38(01):64-9. doi: 10.13213/j.cnki.jeom.2021.20317 (In Chinese).

112. Zhang P, Liu T, Zhang M, Zhang C, Shi Z, Yan F. Comparison of Application of Four Occupational Health Risk Assessment Methods in Electronic Component and Component Manufacturing Enterprises. *Preventive Medicine* (2021) 33(09):928-31. doi: 10.19485/j.cnki.issn2096-5087.2021.09.017 (In Chinese).

113. Xu Q, Cao Y, Wang P, Ren H, Yuan W, Li F, et al. Comparison of Five Occupational Health Risk Assessment Models Applied to Silica Dust Hazard in Small Open Pits. *Preventive Medicine* (2021) 33(09):873-6+83. doi: 10.19485/j.cnki.issn2096-5087.2021.09.003 (In Chinese).

114. Qin R, Tang H, Li M, Liu B, Ceng Q. Comparison of Application of Different Risk Assessment Methods in Dust Hazard Assessment in Gypsum Board Production Enterprises. *Preventive Medicine* (2021) 33(11):1161-5. doi: 10.19485/j.cnki.issn2096-5087.2021.11.021 (In Chinese).

115. Zhang S, Wang R, Tao L, Zhang P, Zou W, Wei H. Application of Improved Comprehensive Index Method in Risk Assessment of Frp Yacht Manufacturing Enterprises. *Chinese Journal of Industrial Hygiene and Occupational Diseases* (2021) 39(2):151-4. doi: 10.3760/cma.j.cn121094-20200221-00070 (In Chinese).

116. Tang S, Shen H, Chen Z, Mao G, Liang J. Comparison of Three Methods Applied in Occupational Health Risk Assessment of Lead Related Posts. *Chinese Journal of Industrial Medicine* (2021) 34(06):543-5. doi: 10.13631/j.cnki.zggyyx.2021.06.024 (In Chinese).

117. Wang TS, Song B, Sun QH, Lin YX, Sun Y, Sun P, et al. Occupational Health Risk Assessment of Benzene, Toluene, and Xylene in Shanghai. *Biomedical and Environmental Sciences* (2021) 34(4):290-8. doi: 10.3967/bes2021.038.

118. Chen W, Huang Y, Chen S, Li H, Li B, Su S. Application Comparison of Three Kinds of Occupational Risk Assessment Methods in Lighting Industry. *Chinese Journal of Industrial Medicine* (2021) 34(03):251-5. doi: 10.13631/j.cnki.zggyyx.2021.03.020 (In Chinese).

119. Wang X, Hu W, Zhang S, Kang N, Wang H, Dong Y, et al. Occupational Dust Hazards and Risk Assessment of Coal-Fired Thermal Power Plants of Different Capacities - China, 2017-2019. *China CDC Weekly* (2021) 3(43):901-5. doi: 10.46234/ccdcw2021.221.

120. Zhao X, Ceng Q, Liu J, Ni Y, Wang X, Gu Q. Application of Five Methods in the Occupational Health Risk Assessment of Workers Exposed to Welding Fumes. *Chinese Journal of Industrial Hygiene and Occupational Diseases* (2021) 39(05):375-8. doi: 10.3760/cma.j.cn121094-20200630-00368 (In Chinese).

121. Wang X, Zhong X, Xie Z, Guan Y, Zhu X, Zhou W. Application of Multi-Risk Assessment Methods in Occupational Health Risk Assessment of Silica Dust Hazards in Workplace of Stone Processing Industry. *Chinese Journal of Industrial Medicine* (2021) 34(03):255-9. doi: 10.13631/j.cnki.zggyyx.2021.03.021 (In Chinese).

122. Zhang Y, Xiao J, Li Z, Chen X, Cai W, Fu H, et al. Application of Two Occupational Health Risk Assessment Methods in Enterprises with 1-Bromopropane Production and Utilization. *China Occupational Medicine* (2021) 48(04):397-401. doi: 10.11763/j.issn.2095-2619.2021.04.007 (In Chinese).

123. Qiu Y, Bian H, Lin B, Lin W, Dai H, Zhang S, et al. Application of Icmm Assessment Model in Occupational Health Risk Assessment of a Metal Surface Treatment Enterprise. *Occupation and Health* (2021) 37(01):20-3. doi: 10.13329/j.cnki.zyyjk.2021.0004 (In Chinese).

124. Niu Y, Zhang L, Liu K, Yu B, Zhang R, Han L, et al. Occupational Health Risk Assessment of Dust in Cement Production Enterprises. *Preventive Medicine* (2021) 33(06):558-62+67. doi: 10.19485/j.cnki.issn2096-5087.2021.06.004 (In Chinese).

125. Gu Y, Wang A, Mao H, Hu X, Leng P, Miao C. Analysis of Occupational Health Risk Assessment Results of Two Ferrous Metal Foundry Enterprises. *Preventive Medicine* (2021) 33(09):924-7+31. doi: 10.19485/j.cnki.issn2096-5087.2021.09.016 (In Chinese).

126. Feng Y, Zhao Y, Gao Y, Zhang H, Liu Y. Application of Different Risk Assessment Methods in Occupational Health Risk Assessment in Sewage Treatment Industry. *Occupational Health and Emergency Rescue* (2021) 39(01):45-9+79. doi: 10.16369/j.oher.issn.1007-1326.2021.01.009 (In Chinese).

127. Yu Z, Zhang C, Liu X, Zhang H. Study on the Application of the Technical Guidelines of Occupational Health Risk Assessment of Hazardous Chemical Factors in Workplace in Lead-Acid Battery Industry. *Jiangsu Journal of Preventive Medicine* (2021) 32(02):167-72. doi: 10.13668/j.issn.1006-9070.2021.02.013 (In Chinese).

128. Xiao Z, Zhou D, Li W, Chang L, Wang N. Occupational Chemical Hazard Risk Assessment of Benzene and Its Analogies in Storage Tank Areas of Petrochemical Enterprises Based on Risk Matrix Method. *Journal of Environmental and Occupational Medicine* (2021) 38(10):1140-4. doi: 10.13213/j.cnki.jeom.2021.21078 (In Chinese).

129. Nie Z. Analysis the Occupational Health Status of Coal Dust in 107 Non-Metallic Mineral Products Enterprises in Sichuan Province and the Applicability of Semi-Quantitative Risk Assessment Model [master's thesis]. Sichuan: Southwest Medical University (2021). doi: (In Chinese).

130. Zhou Z, Su S, Ceng Y. Comparative Study on Common Occupational Health Risk Assessment Methods in a Paint Manufacture. *Chinese Journal of Public Health Engineering* (2021) 20(05):719-23+26. doi: 10.19937/j.issn.1671-4199.2021.05.005 (In Chinese).

131. Mo Z, Lu S, Shao M. Volatile Organic Compound (Voc) Emissions and Health Risk Assessment in Paint and Coatings Industry in the Yangtze River Delta, China. *Environmental pollution* (2021) 269:115740. doi: 10.1016/j.envpol.2020.115740.

132. Xie B, Chang W, Wu K, Guo L, Mei Y. Application of Three Risk Assessment Methods to Noise Risk Assessment in an Automobile Foundry Enterprise. *Chinese Journal of Industrial Hygiene and Occupational Diseases* (2022) 40(04):271-5. doi: 10.3760/cma.j.cn121094-20210109-00036 (In Chinese).

133. Yang C, Nie Z, Tang D, Chen F, Ceng Y, Jia H. Application of Three Semi-Quantitative Risk Assessment Methods in Brick and Tile Enterprises. *Occupation and Health* (2022) 38(10):1297-301+306. doi: 10.13329/j.cnki.zyyjk.2022.0276 (In Chinese).

134. Zhang C, Wang M, Mao G, Liang J, Guo D, Yi G, et al. Comparative Study of Risk Assessment Model and Actual Monitoring in the Exposure Evaluation of Automobile Manufacturers. *Industrial Health and Occupational Diseases* (2022) 48(02):101-4+24. doi: 10.13692/j.cnki.gywsyzyb.2022.02.004 (In Chinese).

135. Bian H, Liao C, Liang S, Qiu Y, Lin W, Su S. Application of Multiple Methods in the Assessment of Occupational Health Risk of N-Hexane in No-Dust Workplaces. *China Occupational Medicine* (2022) 49(01):57-61. doi: 10.20001/j.issn.2095-2619.20222010 (In Chinese).

136. Liu J, Liang J, Mao G, Zhang C, Ye W. A Comparative Study of Semi-Quantitative and Quantitative Models in Risk Assessment of Optical Fiber Manufacturing Enterprises. *Public Health and Preventive Medicine* (2022) 33(04):41-4. doi: 10.3969/j.issn.1006-2483.2022.04.010 (In Chinese).

137. He J, Yin Q, Liao C, Su S. Comparison of Different Semi-Quantitative Risk Assessment Methods Applied in Ammonia and Hydrazine Posts of Power Plants. *Chinese Journal of Industrial Medicine* (2022) 35(03):268-70. doi: 10.13631/j.cnki.zggyyx.2022.03.026 (In Chinese).

138. Jin L, Tang T. Application of Three Occupational Health Risk Assessment Methods in a Coal-Fired Power Generation Enterprise in Western Region of Hainan Province. *Occupation and Health* (2022) 38(12):1590-5. doi: 10.13329/j.cnki.zyyjk.2022.0356 (In Chinese).

139. Zhang L, Sun P, Sun D, Zhou Y, Han L, Zhang H, et al. Occupational Health Risk Assessment of the Benzene Exposure Industries: A Comprehensive Scoring Method through 4 Health Risk Assessment Models. *Environmental Science and Pollution Research* (2022). doi: 10.1007/s11356-022-21275-x.

140. Fan P, Liu K, Zhang L, Wang X, Dong Y, Kang N, et al. Occupational Health Risk Assessment of Silica Dust in Small and Micro Non-Coal Mine Enterprises in Eight Provinces in China from 2018 to 2020. *Journal of Hygiene Research* (2022) 51(02):251-9. doi: 10.19813/j.cnki.weishengyanjiu.2022.02.013 (In Chinese).

141. Guo Q, Li M, Huang D, Zhang Q. Risk Assessment Method and Application of Occupational Hazards in Operation Exposed to Aromatic Mixture Based Pbpk Model. *Chinese Journal of Industrial Medicine* (2022) 35(03):200-4. doi: 10.13631/j.cnki.zggyyx.2022.03.002 (In Chinese).

142. Dong Q, Li J, Liu K, Lu G, Zhao J, Li S, et al. Applicability Analysis of Four Occupational Health Risk Assessment Methods for Jobs Exposed to Silica Dust. *China Occupational Medicine* (2022) 49(02):196-200. doi: 10.20001/j.issn.2095-2619.20224014 (In Chinese).

143. Su S, Liang Z, Zhang S, Xu H, Chen J, Zhao Z, et al. Application of Multiple Occupational Health Risk Assessment Models in Occupation Health Risk Prediction of Trichloroethylene in the Electroplating and Electronics Industries. *International Journal of Occupational Safety and Ergonomics : JOSE* (2022):1-7. doi: 10.1080/10803548.2021.2022956.

144. Shi T, Wang Y, Wang S, Yang Y, Lan Y, Cui F, et al. Application Research on Occupational Health Risk Assessment Technology of Dust in Brick and Tile Manufacturing Industry. *Occupational Health and Emergency Rescue* (2022) 40(03):298-304. doi: 10.16369/j.oher.issn.1007-1326.2022.03.008 (In Chinese).

145. Ye W, Mao G, Liang J, Liu J, Zhang C, Chen Z. Study on Applicability of Five Occupational Health Risk Assessment Methods in Paint Position of Bus Maintenance Company. *Occupation and Health* (2022) 38(11):1446-9. doi: 10.13329/j.cnki.zyyjk.20220429.007 (In Chinese).
